# Consensus clustering methodology to improve molecular stratification of non-small cell lung cancer

**DOI:** 10.1038/s41598-023-33954-x

**Published:** 2023-05-12

**Authors:** L. Manganaro, S. Bianco, P. Bironzo, F. Cipollini, D. Colombi, D. Corà, G. Corti, G. Doronzo, L. Errico, P. Falco, L. Gandolfi, F. Guerrera, V. Monica, S. Novello, M. Papotti, S. Parab, A. Pittaro, L. Primo, L. Righi, G. Sabbatini, A. Sandri, S. Vattakunnel, F. Bussolino, G.V. Scagliotti

**Affiliations:** 1 aizoOn Technology Consulting S.R.L, Torino, Italy; 2 Medical Oncology Division at San Luigi Hospital, Department of Oncology, University of Torino, Orbassano (TO), Italy; 3 Department of Translational Medicine, Piemonte Orientale University, Novara, Italy; 4 Center for Translational Research on Autoimmune and Allergic Diseases-CAAD, Novara, Italy; 5 Department of Oncology, University of Torino, 10060 Candiolo, Italy; 6 Candiolo Cancer Institute-IRCCS-FPO, 10060 Candiolo, Italy; 7 Division of Thoracic Surgery at AOU San Luigi, Department of Oncology, University of Torino, Orbassano (TO), Italy; 8 Division of Thoracic Surgery at AOU Città della Salute e della Scienza, Department of Surgical Sciences, University of Torino, Torino, Italy; 9 Pathology Division at AOU Città della Salute e della Scienza, Department of Oncology, University of Torino, Torino, Italy; 10 Pathology Division at AOU San Luigi, Department of Oncology, University of Torino, Orbassano (TO), Italy

**Keywords:** Non-small-cell lung cancer, Machine learning, Transcriptomics

## Abstract

Recent advances in machine learning research, combined with the reduced sequencing costs enabled by modern next-generation sequencing, paved the way to the implementation of precision medicine through routine multi-omics molecular profiling of tumours. Thus, there is an emerging need of reliable models exploiting such data to retrieve clinically useful information. Here, we introduce an original consensus clustering approach, overcoming the intrinsic instability of common clustering methods based on molecular data. This approach is applied to the case of non-small cell lung cancer (NSCLC), integrating data of an ongoing clinical study (PROMOLE) with those made available by The Cancer Genome Atlas, to define a molecular-based stratification of the patients beyond, but still preserving, histological subtyping. The resulting subgroups are biologically characterized by well-defined mutational and gene-expression profiles and are significantly related to disease-free survival (DFS). Interestingly, it was observed that (1) cluster B, characterized by a short DFS, is enriched in KEAP1 and SKP2 mutations, that makes it an ideal candidate for further studies with inhibitors, and (2) over- and under-representation of inflammation and immune systems pathways in squamous-cell carcinomas subgroups could be potentially exploited to stratify patients treated with immunotherapy.

## Introduction

Lung cancer causes 1.8 million deaths per year and carries the greatest economic burden of all cancers, costing €18.8 billion per year in Europe alone^1,2^.

In this context, the vast majority of lung cancer (~ 85%) are classified as non-small cell lung cancer (NSCLC), with the major histologic subtypes being adenocarcinomas, squamous carcinomas and, to a lesser extent, large cell carcinomas, whereas small cell lung cancer (SCLC) accounts for the remaining 15%. While clinical and imaging features can help the clinician distinguish NSCLC from SCLC, histopathologic features and immunohistochemical markers are required to make further subtyping^3^.

Lung cancer, and in particular NSCLC, has benefited from the clinical implementation of precision medicine, since it has become clear that the introduction of routine molecular testing, which might lead to targeted therapies, in clinical practice has significantly contributed to an initial decrease of patients’ mortality, with 5 year-survival rates near to 22% nowadays, as compared to 17% few years ago^2^. In recent years, the renewed interest in agents able to relieve the immunosuppression state observed in many human cancers, namely the immune-checkpoint inhibitors^4^, has paved the way to further improvements. Despite these achievable survival benefits, the outlook for the majority of patients is still unsatisfactory and more collaborative and comprehensive approaches are needed.

Tumour evolution is the result of genomic instability caused by somatic mutations, chromosomal rearrangements, copy number alterations and epigenetic changes, resulting in loss of tumour suppressor genes and activation of oncogenes that disengage the affected cells from their regulatory cycles^5,6^. Understanding this progression is the unifying goal of much cancer research which aims at improving patients’ management strategies^7–9^.

The recent advances in artificial intelligence (AI) and machine learning (ML) research^10^, combined with cheaper sequencing costs enabled by modern next-generation sequencing (NGS) technology, brought to a new era of bioinformatics. In this renovated context, scientists are challenged to analyse huge amounts of data to unveil the molecular mechanisms underlying complex pathologies such as cancer. One of the major aims of such analyses is represented by disease subtyping, which consists in stratifying patients into different clusters based on similarities and differences between their molecular profiles. Several clustering models designed for single- and multi-omics data have been developed and can be roughly divided in three main categories: variance-based, similarity-based, and network-based models. All models must face the dimensionality issue deriving from the high number of genomic features, which are typically orders of magnitude larger than the number of samples in the dataset^11^.

Variance-based models implement an initial step of dimensionality reduction in which raw data is projected in a lower-dimensional subspace; i.e*.*, which dimensions catch most of the variance of original data. Among these models, it is worth mentioning iCluster^12^ and MOFA^13^, with their subsequent enhancements iClusterPlus^14^, iClusterBayes^15^ and MOFA+ ^16^ respectively, JIVE^17^ and intNMF^18^. Similarity-based models solve the dimensionality issue by applying kernels to the original data, generating a similarity matrix between patients through well-known methods such as spectral clustering^19^. The most known of such models are SNF^20^, ANF^21^ and SRF^22^. Finally, network-based models build the network or multi-network of interactions between genes which is then exploited to get meaningful features for clustering. This is for example the case of NBS^23^ and SALMON^24^.

A major and common issue to all the models is that they rely on many parameters which are hard to be tuned when solving an unsupervised learning task, whereas they may strongly affect the resulting clustering. Moreover, each of the above-mentioned categories are focused on different aspects of the data, depending on the structure and the metrics of the model itself, and focusing on a single aspect may be limiting for finding general subgroups. The final choice of the model and parameters is often guided using cluster validity indexes (CVI)^25^, such as the Silhouette index, which are also not conclusive, since each CVI is biased toward models, utilising similar metrics of the adopted CVI^26^. All these features are particularly critical in the case of non-small cell lung cancer (NSCLC), which is a highly heterogenous tumour^27^, with molecular profiles characterised by a large biological variability leading to poor performance of the prognostic models^28,29^.

To address all these issues, we adopted a cutting-edge ensemble learning approach, known as consensus clustering^30^, which already proved excellent performance with other multi-omics datasets^31^ and it is here applied for the first time to NSCLC, revealing well characterised molecular subtypes with prognostic implications. Such approach consists in training several diverse and independent clustering models (learners) and integrating their results through a voting process, deriving a final clustering where labels are those with the most support from the community of independent learners. It should be noted that the proposed approach has a general validity and might be easily customised and adapted to other cancer types.

The present study is partly based on the early stage (I-IIIa) sub-group of NSCLC patients recruited in the prospective observational clinical PROMOLE study^32^ (which is part of a wider research project named DEFLeCT) designed to correlate follow-up, clinical and pathological information with molecular features including mutations, translocations, copy number variations and gene expression profiles respectively collected by exome and bulk-RNA sequencing (see also Supplementary Fig. 1 and 2). To maximise the number of samples considered and to ensure the generalisation capability of the models and the stability of the derived molecular profiles, the PROMOLE datasets have been integrated with the public gene expression and mutational profiles made available by The Cancer Genome Atlas (TCGA)^33^.

In summary, relying on a cutting-edge ensemble learning approach and applied to well-established (TCGA) as well as newly acquired (PROMOLE) data, the present study increases the current understanding of NSCLC, providing a novel and robust molecular-based stratification of such disease, with well-defined molecular profiles that represent a potential signature against which to test new patients and with prospective implications in immunotherapy.

## Results

### Major histological subtypes are well identified

**Figure 1:**
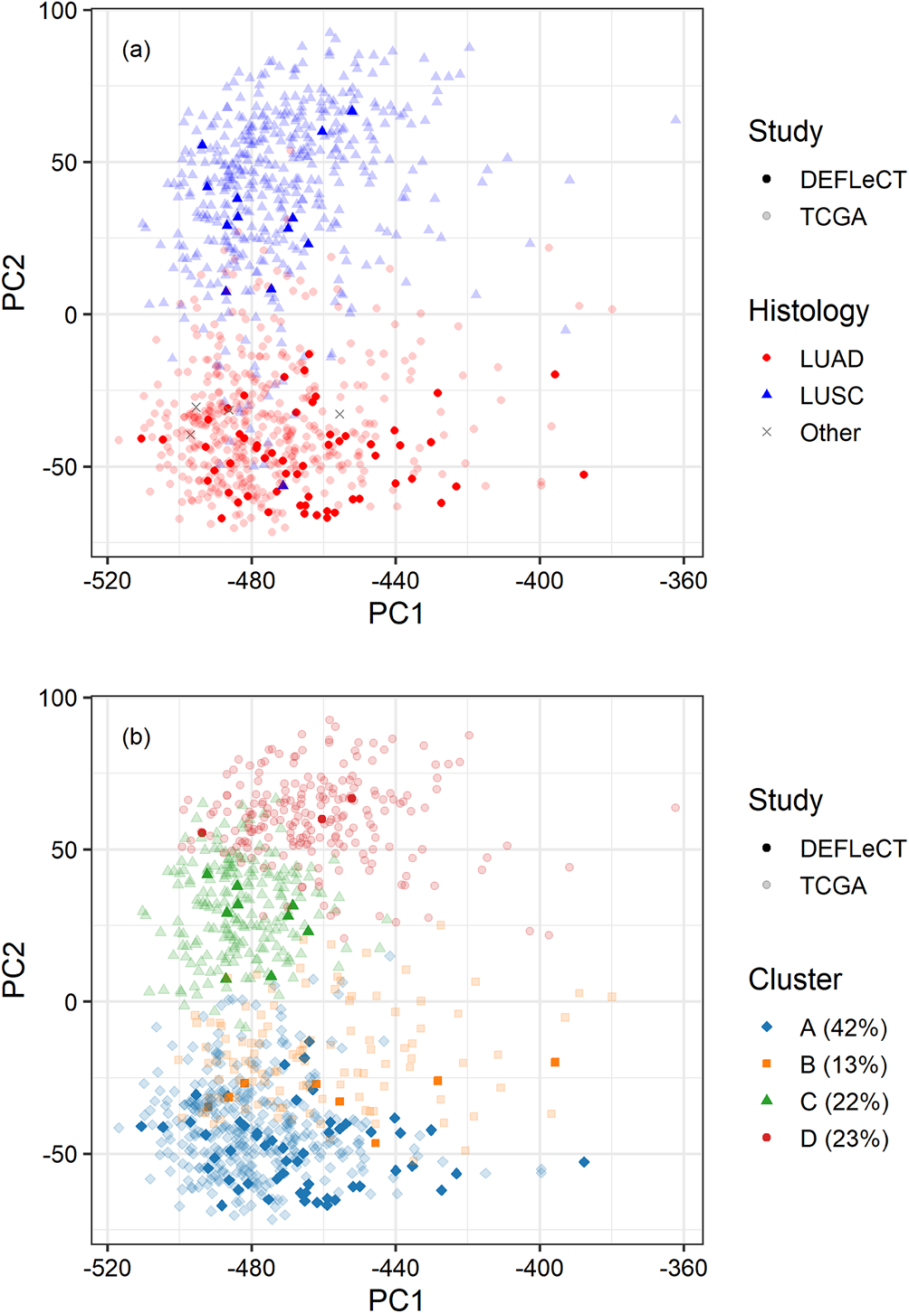
First two principal components of the gene expression profiles of TCGA (transparent) and DEFLeCT (alias PROMOLE) (opaque) samples obtained via PCA of the log2-scaled TPM. (**a**) Colours and shape distinguish LUAD (red dots) and LUSC (blue triangles), not jet available histology of DEFLeCT samples are marked with a grey cross (x). (**b**) Colours and shape indicate the 4 molecular subgroups identified via consensus clustering; relative abundance of each subgroup is reported in the legend.

The gene expression profiles of PROMOLE samples (n = 81) were jointly normalised and integrated with TCGA data (n = 889) (see “Methods”). The resulting dataset had negligible batch-effect (Fig. 1 and Supplementary Fig. 3) and it was suitable for the adoption of machine learning methods, which benefit from a higher number of samples. As shown in Fig. 1a, the major histological subtypes of NSCLC, namely lung adenocarcinomas (LUAD) and lung squamous cell carcinomas (LUSC), present well characterized gene expression profiles that are effectively separated by conventional dimensionality reduction techniques such as principal component analysis (PCA). Unsupervised and supervised single-omics machine learning approaches have been applied and resulted in 95% and 97% accuracy in recognising LUAD/LUSC subtypes respectively.

### Consensus clustering reveals novel molecular-based subtypes

**Figure 2:**
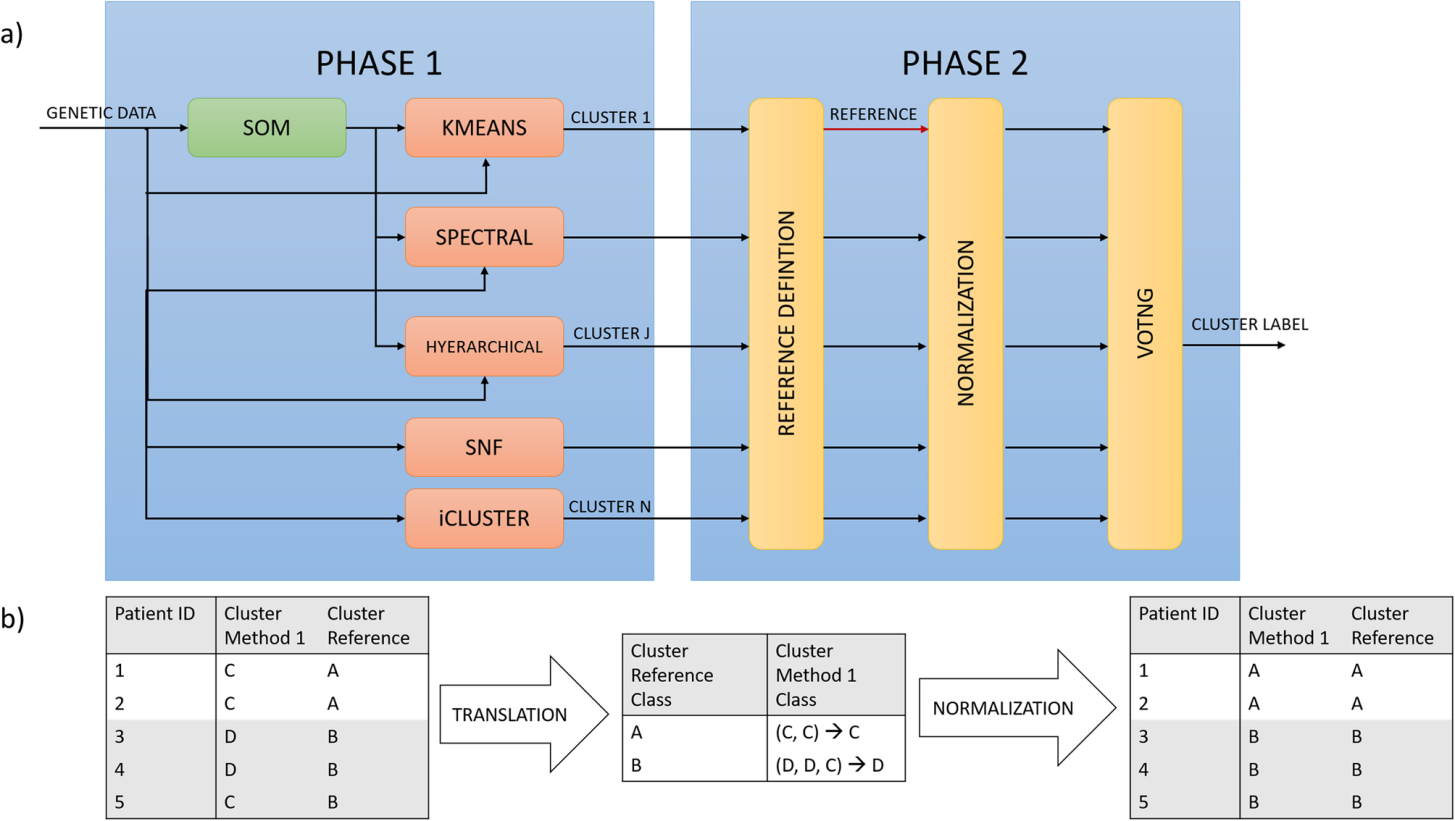
(**a**) Workflow of consensus clustering algorithm. In phase 1, many independent clustering algorithms (learners) are trained separately. In phase2, a reference for labels is chosen, clustering labels are aligned to the reference nomenclature, final labels are derived via majority voting. (**b**) Detail of the consensus clustering phase 2 normalization procedure. Each clustering method association is firstly translated with respect to the reference clustering method (employing the most common labels among the clusters defined by the reference), and then reported in the normalized reference nomenclature.

**Figure 3:**
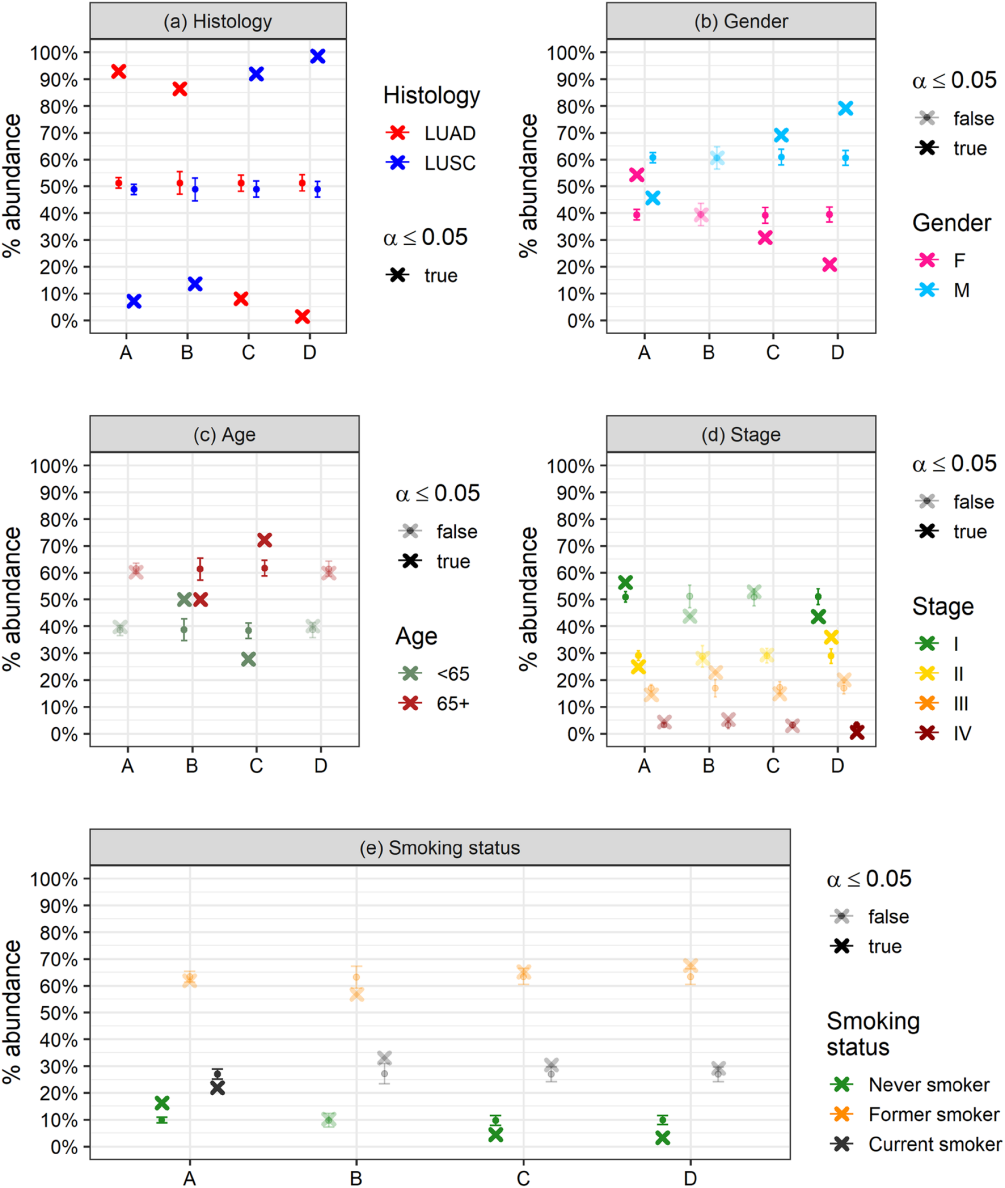
Comparison between observed (cross) and expected (dots and error bar) relative abundances of the main histological subtypes and clinical variables in the 4 molecular subgroups. Transparency refers to significance according to normal Z-test (transparent: not significative, opaque: significative *p* < 0.05). (**a**) Histological subtypes; (**b**) gender; (**c**) age grouped as under-/over- 65; (**d**) clinical staging (grouped in 4 levels); (**e**) smoking status.

Using the above-mentioned gene-expression profiles, we adopted an ensemble learning approach to investigate whether novel biological subgroups could be found by consensus clustering (see “Methods”). Such approach, which is designed in two phases (Fig. 2), integrates the independent clusters resulting by the training of many diverse and well-established clustering models (phase 1), namely K-Means clustering, spectral clustering, hierarchical clustering, iClusterPlus, and similarity network fusion, the first three both directly applied on data and coupled with self-organising maps (for detailed description and references see “Methods”), through a voting process (phase 2). The final labels assigned by consensus clustering are those supported by many independent learners and have the major advantage of being more stable and general with respect to that obtained by each independent clustering model. The choice of the optimal number of clusters considers several aspects: firstly, the structure of the resulting clustering, which should preserve the separation between histological subgroups; secondly, the values of CVIs such as Silhouette index, despite care is needed to avoid biased choices; and finally, the biological characterisation of the resulting subgroups. In this case, cluster analysis indicated the presence of 4 subgroups, as represented in Fig. 1b (details on independent and consensus clustering labels are reported in Supplementary Table 1). This is supported by all the above-mentioned indicators. In particular, it was the optimal value resulting from Silhouette index (see Supplementary Table 1 and Supplementary Fig. 4). Moreover, it was the configuration which best matched the histological structure: specifically, subgroups A and B are composed mostly by LUAD (which are significantly over-represented with an abundance of 92.6% and 86.1% respectively), while subgroups C and D cover the vast majority of LUSC cases (with abundances of 91.6% and 98.6% respectively), as shown in Fig. 3a (and Supplementary Table 1). Finally, the 4 emerging subgroups presented distinct and well-characterised molecular profiles from both gene expression and mutational perspectives, as discussed in the following sections.

### Relationship between molecular subtypes and disease-free survival

**Figure 4:**
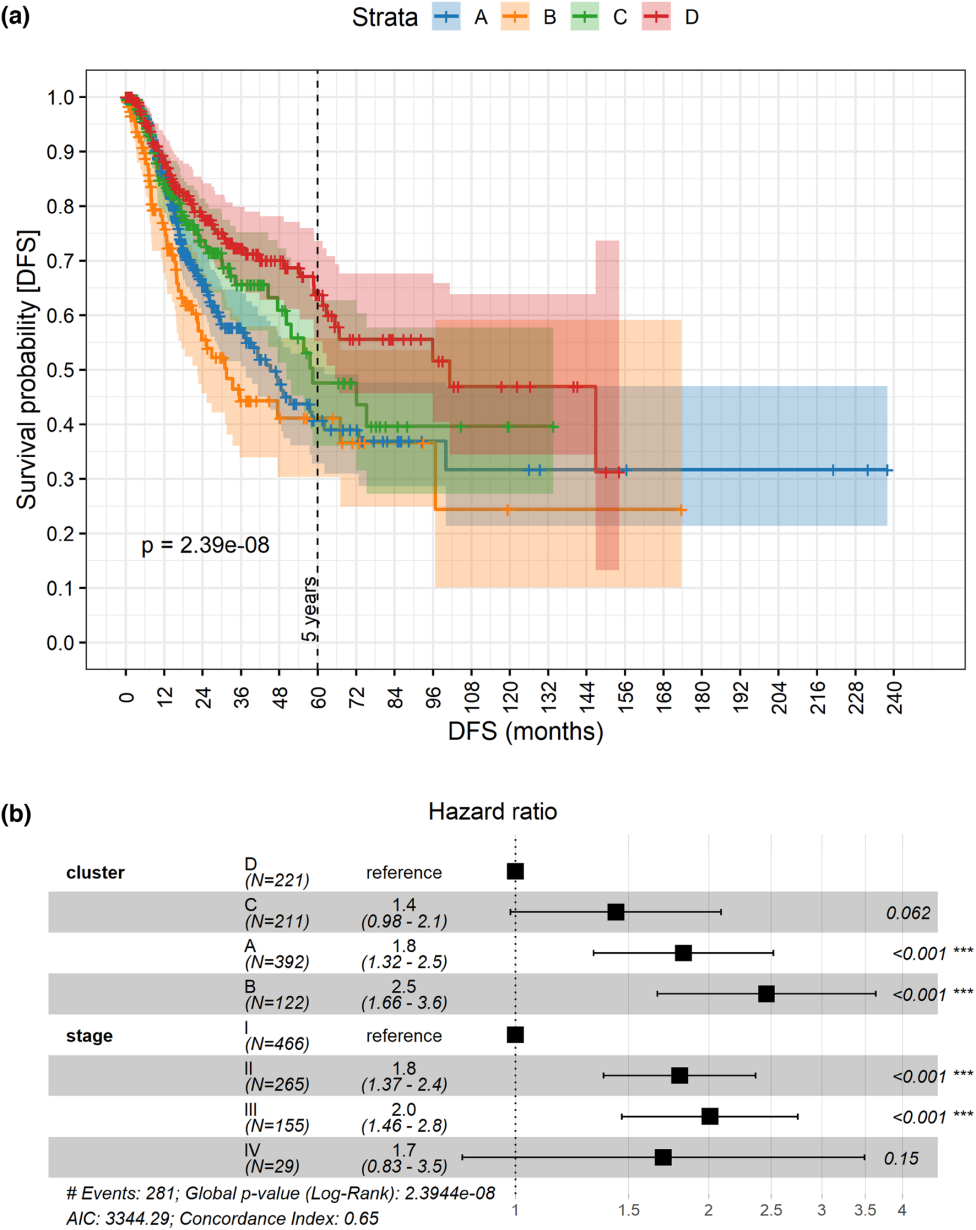
(**a**) Kaplan–Meier survival curves and confidence intervals representing the DFS of the 4 molecular subgroups with the associated log-rank *p*-value. (**b**) Forest plot representing the hazard ratios and significance of the subgroups and clinical stage (the only significant clinical confounder) computed via Cox regression model.

We computed Kaplan–Meier curves representing the disease-free survival (DFS) of each identified subgroup (Fig. 4a). DFS was available for 881 out of the 889 TCGA patients, and for 73 out of the 81 PROMOLE patients. The curves are well separated, with a log-rank *p*-value of $2.39\times {10}^{-8}$. As expected, confidence intervals became broader with increasing time due to reduced residual statistics. In addition, we fitted a Cox proportional hazard model (Fig. 4b) to evaluate the clusters hazard ratios (HR). We first tested all the available clinical features in a Cox model, using the emerging subtypes as regressor, and clinical staging was found as the only significant confounder among the tested variables (see “Methods”). Interestingly, histology did not appear to be significative in the Cox regression because its effect was masked by the finest grouping and stronger impact on the DFS carried by the identified molecular subgroups. The HR analysis indicated significative differences in DFS between the four groups: in particular, cluster D was significantly associated with a better outcome, while clusters A and B were characterised by a significantly increased risk of recurrence (*p* < 0.001). Overall, survival analysis proved that the identified subgroups were characterised by distinct clinical outcomes in terms of DFS. To provide a clinical characterisation of the subgroups, we tested the relative abundances of the main clinical variables for each emerging cluster with χ^2^ test (see Supplementary Table 2) and with Z-tests against their respective null models (Fig. 3). Null models were built through bootstrap resampling of the consensus clustering labels over the whole population. Clinical characterisation showed that: clusters C-D and A were characterised by a significant over-representation of males and females respectively (Fig. 3b); cluster B, which is the shortest living in terms of DFS, was characterised by an over-representation of under 65, who were instead under-represented in cluster C (Fig. 3c); cluster D, which was the longest living in terms of DFS, was characterised by under-representation of stage IV patients and over-representation of stage II, while cluster A has an over-representation of stage I (Fig. 3d); finally, non-smokers were under-represented in clusters C-D, and over-represented in cluster A, which was also characterised by a under-representation of smokers (Fig. 3e). However, some of those differences may be related to some kind of selection bias.

### Different molecular subtypes show specific transcriptomic and exomic signatures

**Figure 5:**
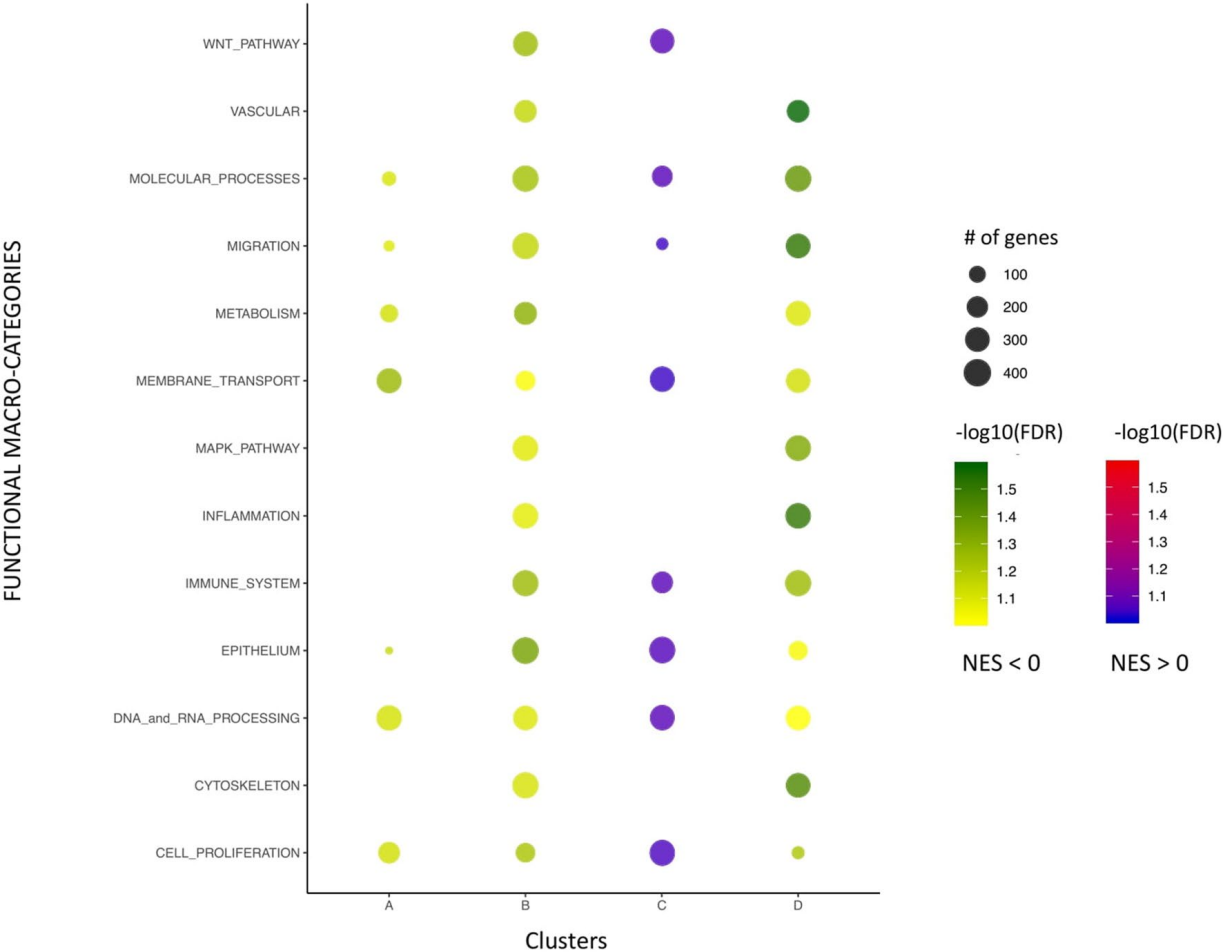
Dot plot of the most representative functional categories (rows) that are significantly enriched in at least one of the 4 molecular subgroups (columns) with respect to the others according to GSEA analysis. Colour scale indicates the value of the FDR value for positive and negative NES, dot size is proportional to the number of genes associated to each macro-category.

To investigate the biological meaning of the identified molecular subtypes, we applied a gene-set enrichment analysis (GSEA) to investigate possible enriched functions associated to each of the four consensus clusters (see “Methods”). In so doing, we analysed the enrichment of Gene Ontology, KEGG Pathways and Hallmark of Cancer gene sets from the MSigDB database^34^ for each of the four clusters separately, versus all the remaining samples. Results are reported in Fig. 5. We focused our attention on results associated with a false discovery rate (FDR) < 0.1 and we defined a set of thirteen, manually curated, selected major functional macro-categories, which allowed us to group and better summarize related functional classes found over-represented.

Overall, cluster C appeared to be the most enriched in functions compared to the rest of the samples (-normalised enrichment score- NES > 0). Details of the most representative enriched pathways can be found on Supplementary Fig. 5 and 6. Clusters A, B and D were instead mainly associated with functions that were negatively regulated in the samples associated with them compared to all the others (NES < 0). In general, cluster B and D were more prone to show functional enrichments with respect to cluster A and C, of which results are associated with relatively few molecular sets.

MEMBRANE_TRANSPORT, EPITHELIUM and MIGRATION related terms resulted to be commonly modulated in all the four cluster, even if positively in cluster C or negatively in clusters A, B and D. In addition, also the functional macro-categories DNA_and_RNA_PROCESSING and CELL_PROLIFERATION were among the top-enriched ones, suggesting a common dysregulation of proliferative genes in all the samples we profiled. CYTOSKELETON, VASCULAR, MAPK pathways genes and general IMMUNE_SYSTEMS/INFLAMMATION gene sets are instead significantly linked only with cluster B and D. Several METABOLISM-associated gene sets resulted significantly negatively enriched only in cluster A, B and D. Gene sets correlated with WNT_PATHWAY were specifically associated only to cluster B and C. The MOLECULAR_PROCESSES macro-category groups several gene sets associated with the major cellular functions (e.g*.* signal transduction, protein expression and translation control, regulation of protein activity; see Supplementary Materials) resulted enriched in the four comparisons.

The surprising observation that NES was positive just in cluster C, which represents a fraction of LUSC, finds evidence in literature. For instance, it was reported that members of WNT pathway, cell cycle, MAP kinases, and DNA/RNA processing genes (see e.g. DNA repair, spindle organization, minichromosome maintenance protein) were significantly overexpressed in LUSC compared to LUAD^35,36^. Moreover it has been reported that subtypes of LUAD and LUSC respectively showed decreased and elevated immune cell expression^37^, which might reflect the enrichment of cluster C of immune-related genes. Generally, it has been established that marked differences of immune-response related genes in LUAD and LUSC^38,39^ could explain different responses to immune checkpoint inhibitors^40^.

**Figure 6:**
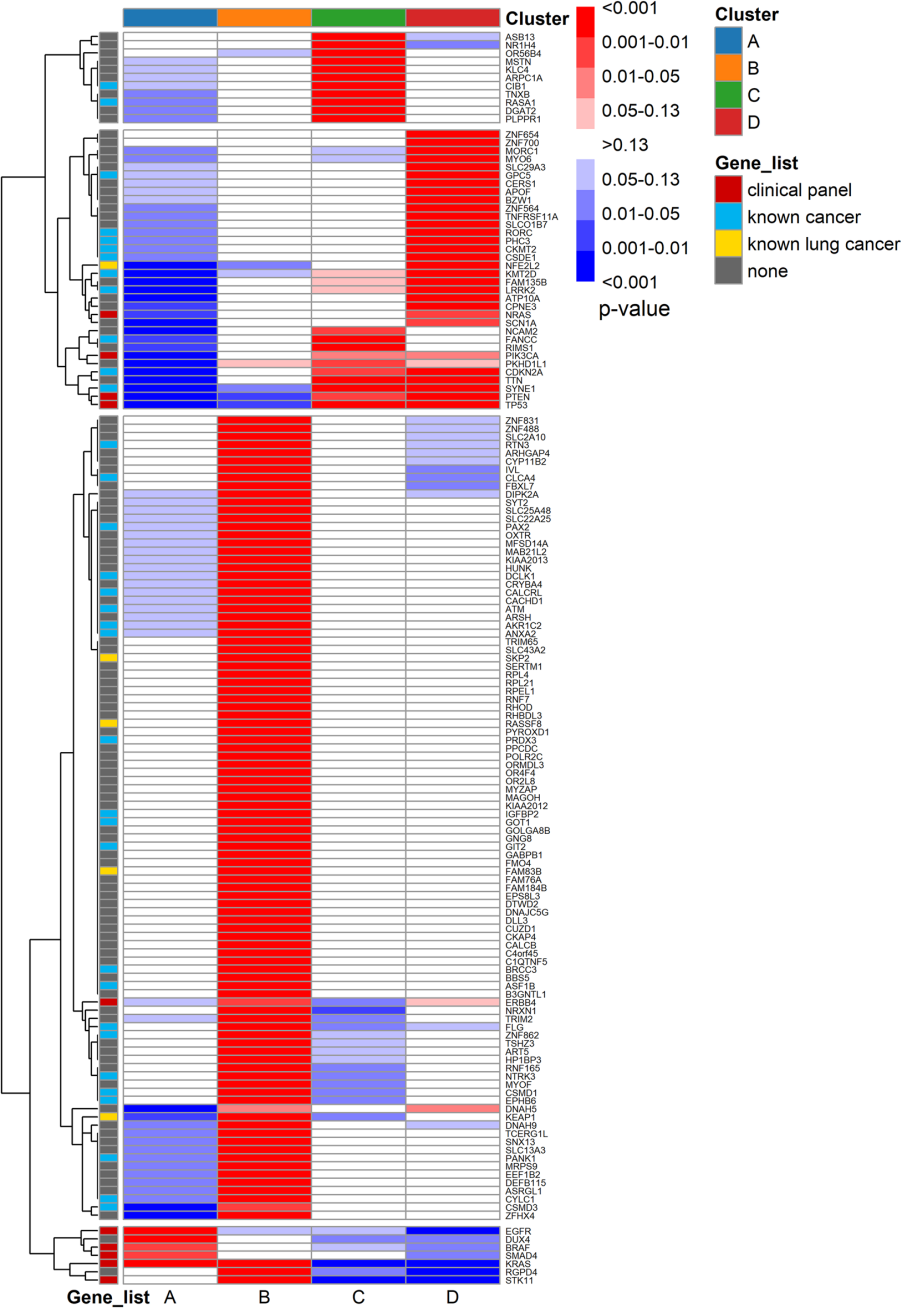
Heatmap of the 150 most representative genes (rows) quantifying over- and under-represented mutations of 4 molecular subgroups (columns). Colour scale indicates normal Z-scores grouped by *p*-value classes. Genes are rearranged by hierarchical clustering to facilitate pattern recognition. Genes are flagged via colour annotations if belonging to meaningful selected lists, prioritised as follows: clinical panel (red), lung-cancer related genes (yellow), cancer-related genes (cyan), other (grey).

We also characterised the mutational landscape of the identified molecular subgroups by quantifying over- and under-represented mutated genes of each cluster through Z-scores analysis (Fig. 6). To increase readability and interpretability of the results, significative genes have been flagged based on their presence in selected lists, namely: gene panel designed for detailed molecular analysis of PROMOLE study, genes with known association with lung cancer and genes with known associations with cancer in general. The last two lists have been derived by comprehensive analysis of the GeneCards database.

From a mutational perspective, the identified subgroups presented well-defined and recognisable genetic alterations. First of all, clusters A and B shared major features widely associated with LUAD, such as *KRAS* mutations^41^ but also showed well distinct features, namely: cluster A was characterised by over-represented mutation of *EGFR* oncogene and interestingly a significative (*p* < 0.001) under-representation of *KEAP1* and *NFE2L2* mutations, which has already been found to be mutually exclusive with *EGFR*^42^. *KEAP1* mutations, which has been identified as a potential prognostic factor in resected NSCLC^43^, were instead over-represented in cluster B, which we found to be as the one with the poorest outcome in terms of DFS (Fig. 4a). Cluster A also exhibited over-represented (*p* < 0.01) mutations of well-known *BRAF* and *SMAD4* genes. Cluster B was characterized by over-represented mutations in established or candidate tumour suppressor genes such as *STK11*^44^ and *RASSF8*^45^ and oncogenes such as *SKP2*^46^ and *FAM83B*, which participates in the EGFR signalling pathway and may activate both the EGFR itself and downstream RAS/MAPK and PI3K/AKT/TOR signalling cascade^47^. Moreover, cluster B was characterised by genetic alterations of *ERBB4*, and DNA-repair genes such as *ATM* and *PANK1*, where the first is co-regulated by the tumour suppressor *TP53*, while the second functions as a regulator of a wide variety of downstream proteins, including tumour suppressor proteins p53 and *BRCA1*, checkpoint kinase *CHK2*, checkpoint proteins *RAD17* and *RAD9*, and DNA repair protein *NBS1*. Cluster C and D shared major features of LUSC such as *PIK3CA* and tumour suppressor *TP53*, *PTEN* and *CDKN2A* mutations, which were instead significatively under-represented in cluster A and B. Cluster D was well characterised by significant (*p* < 0.001) over- and under- representation of *NFE2L2* and *EGFR* mutually exclusive mutations respectively and *NRAS* over-representation while cluster C exhibited unique over-representation of mutated *RASA1*, which SH2 domain has been identified as a probably oncogenic^48^, and *CIB1* and *FANCC*, which are involved in post-replication DNA repair^49^. It is important to note that other relevant mutations typically associated with lung cancer (such as *ALK*, *ROS1*, *RET*, *FGFR1*, *ERBB2*, etc.) have been found with frequencies compatible with previously published data, but without a significant difference between subgroups.

## Discussion

In clinical practice, histological classification of lung cancer remains one of the cornerstones of the diagnostic work-up coupled with the assessment of the extent of the disease that is strictly related to survival probability^50^.

As the mechanisms of tumour onset and progression are not restricted to a single molecular level, but rely on a huge number of molecular networks, which in turn sustain intercellular circuits, the detection of groups of patients showing similar characteristics across different omics is a key issue to enable personalized medicine, which aims to offer patients a treatment adapted to the characteristics of their tumours^31^. Bioinformatics approaches are clearly advocated to address this complexity and the clinical value of identifying similar characteristics across different omics is at the root of the development of new computational methods implementing different strategies to analyse several omics datasets simultaneously.

In this context, the main novelty and strength of our approach lies in the effective application of an ensemble learning algorithm to clustering of molecular profiles, that combines multiple state-of-the-art and well-accepted models (such as iClusterPlus and SNF), which are then aggregated through a begging-like majority voting strategy. Ensemble methods, in fact, have been specifically designed to reduce the three main sources of error in machine learning (i.e*.* noise, bias and variance) with respect to traditional single-model approaches, and have the major advantages of resulting in more accurate and especially more stable and robust outcomes. This is crucial in the case of such a multivariate problem as omics data analysis, typically characterised by the so-called *curse of dimensionality*, i.e*.* datasets with a number of features (genes) way above the number of samples (patients). Moreover, ensemble learning may be used to capture both linear and non-linear emerging patterns within data by exploiting different classes of models.

On the other hand, the main limitations of such approach lie, at first, in an intrinsic reduction of interpretability, which increases the effort needed to get a reliable characterisation of the emerging groups, since the relation between inputs and outputs is masked by an increased complexity with respect to traditional approaches. An option to overcome such limitation would be the adoption of eXplainable Artificial Intelligence (XAI) algorithms as proposed by Sabbatini and Manganaro^51^, despite research on this topic is still in its early stage and they are not always applicable to the case of unsupervised learning. Secondly, for the sake of completeness, it is worth mentioning that ensemble methods are by design computationally expensive, so that computing time is significantly increased with respect to single-model applications. This may be limiting for real-time applications; however, it is not the case of this work.

In the last 20 years the detection of specific gene mutations/rearrangements/amplification in NSCLC have been associated with specific targeted therapies which significantly impacted on survival expectations in any stage of lung cancer^52^. Some gene expression signatures to predict NSCLC outcome have been identified^53–55^, but poorly characterized in relationships to the histological subtypes LUAD and LUSC. In this context, the PROMOLE study and the proposed approach represents a further step, well-integrated with what has already been explored in MATCH^56^ or BATTLE-1 and 2^57^ or in the European Proof-of-Concept Therapeutic Stratification Trial of Molecular Anomalies in Relapsed or Refractory Tumors for paediatric tumours (ESMART) trial platform^58^ in which tumour biopsy specimens from adult or paediatric patients are sequenced and used to match patients to relevant treatment arms under defined study protocols.

Our findings have identified four distinct NSCLC subtypes, each associated with unique clinical and molecular features that represent a potential signature against which to test new patients. Such grouping is well supported by a clear relation with DFS, which results in significantly separated survival curves, and which is effectively proved by Cox regression analysis. In fact, it has to be emphasised that the contribution of histology in such analysis is totally masked by stronger impact of the identified molecular subgroups, that overwrite the histological signal. However, it is worth clarifying that the data presented here has to be considered an exploratory cohort that need validation through a confirmatory cohort matched for the major clinical and pathological characteristics to make robust our preliminary observation. As such, while it is unlikely the use of the four clusters as stratification factor for the next generation of clinical trials, they could predict the response to specific treatment such as immunotherapy or targeted therapies.

The clinical correlate of the data presented indicates that the four different identified clusters are summarising what was already known in terms of prognostic information of specific gene mutations and it is questing for the inclusion of molecular information in the next edition of the TNM classification. It should be noted that none of the PROMOLE patients received specific targeted treatments and, consequently, the prognostic impact on DFS is prevalent. With the widespread advent of immunotherapies, it is progressively evident that some genomic correlates, independently from the histological subtypes, are negatively modulating the therapeutic effect of such novel therapies, such for instance STK11 mutation. Beyond shaping the morphological classification of NSCLC by specific genomic alteration in generating a deeper distinction between LUAD and LUSC, there were also potential useful information derived by different clustering of sets of genes leading to a candidate DNA damage-related gene signature associated with an enrichment for immune-related genes in cluster C, making more likely a better response to immunotherapies as already seen in earlier trials with nivolumab^4^. Moreover, it should be noted that the differences between cluster C and D, both belonging to LUSC group, are particularly impressing. Cluster D shows a much better DFS score compared to cluster C and it is characterized by completely different regulation of gene pathways. For instance, inflammation and immune systems gene pathways are over-represented in cluster C compared to D. Although we do not have any experimental evidence linking patient DFS with expression of genes related to immune defence, we can speculate that our consensus cluster analysis might be able to separate LUSC with high level of inflammatory cytokines and much more infiltrated by immune cells, characterized by better DFS, and LUSC less immunoreactive (*i.e.* cold) with a worse prognosis. If this hypothesis will be verified it could be exploited to stratify patients treated with immunotherapy. Additionally, other insights from our classification could open new therapeutic perspectives. In particular, cluster B, characterized by a short DFS, is enriched in KEAP1 and SKP2 mutations. Interestingly, it has been recently reported that small molecule inhibitors of NRF2 pathway sensitize cancer cells carrying KEAP1 mutants to chemotherapy^59^ and that inhibition of SKP2 sensitizes lung cancer cells to paclitaxel^60,61^. Furthermore, LUAD and LUSC classification based on gene expression indicates differential biological mechanisms of cancer onset and progression that in further studies could be mirrored in clinical aspects of NSCLC tumours that stem from abrogated evolutionary signalling and its downstream outcomes.

The definition of four clusters, characterized by different mutational and transcriptomic profiles, opens the question of what are the molecular determinants and pathways that determine the difference between the groups in terms of DFS, that we partly tried to address with the present preliminary study. However, even within the limits of current preclinical models such as cell lines or patient-derived xenografts that cannot recapitulate the role of the microenvironment, the comparative evaluation between the clusters defined in this study and the transcriptomes of preclinical models of NSCLC and the subsequent genetic or pharmacological manipulation with approaches of gain- or loss-of-function could help to identify molecular pathways drivers and new genetic vulnerabilities that explain the different clinical outcome. Depending on the resources available, these experiments will be part of our future research plan.

## Methods

### Acquisition protocol and sample processing

Normal and tumours tissues were collected from 81 resectable patients having histologically confirmed stage IB-IIIA NSCLC and enrolled in a prospective observational clinical trial (PROMOLE) approved by the Ethical Committee of the hospital AOU San Luigi Gonzaga (Orbassano, I) (protocol n.14057 approved in September 28, 2018, n.73/2018) and conducted in accordance with the Declaration of Helsinki. Written informed consent was collected for each participant. At the time of surgical resection, a portion of the tumoral and normal tissue isolated at least 2 cm far from the macroscopical border of the cancer lesion were collected and stored in RNAlater (Thermo) at − 80 °C for subsequent DNA and RNA extraction. Genomic DNA and RNA extractions were respectively performed by Maxwell-RSC Tissue DNA and Maxwell-RSC simply RNA tissue kits (Promega) following manufacturer’s instruction. From RNA samples residual DNA was removed by treatment with RNase-Free DNase Set (QIAGEN). Quality and quantification of nucleic acids was assessed by 2100 Bioanalyzer (Agilent) and Qubit assays (Thermo).

Exome-capture was performed using the SureSelectXT Human All Exon Kit, according to the manufacturer’s instructions (Agilent Technologies). Whole-exome sequencing was performed with the Illumina NovaSeq 6000 (Illumina Inc.) platform with a depth of 35 M and 70 M paired-end reads for normal and tumour samples respectively. In order to identify tumour-specific mutations, we compared each tumour sample to the corresponding normal DNA from normal tissue.

Library was generated with TruSeq® Stranded mRNALibrary (Illumina) and sequenced on a NextSeq 500/550 HighOutput Kit v2.5 flow cell (Illumina), obtaining a mean of 20 million 35–151 bps single-end reads per sample.

### Exome bioinformatics pipeline

After initial quality check^62,63^, NGS sequencing of PROMOLE exomes have been processed through a bioinformatic pipeline in order to determine the mutational landscape of NSCLC patients. In the present study, we adopted the workflow of the IDEA pipeline^64^ which allows to identify: single nucleotide variants (SNV), insertions or deletions (INDEL) and gene copy-number alterations (CNA). The parameters of the pipeline have been tuned in order to minimise the batch-effect with respect to TCGA data and ensure integrability of the two datasets. The results of somatic variant calling have been benchmarked against that of the well-established Genome Analysis ToolKit (GATK) Mutect2^65^ and found to be coherent. Annotation of SNV and INDELS is based on the Catalogue Of Somatic Mutations In Cancer (COSMIC) (https://cancer.sanger.ac.uk/cosmic).

### Transcriptome bioinformatics pipeline

After initial quality check^62,63^ and processing with Cutadapt^66^, RNA-seq data have been processed to calculate the gene expression levels following the workflow described by Korpelainen^67^. Reads alignment to hg38 reference and assembly has been implemented with STAR^68^ and RSEM^69^ (the same tool used in the legacy workflow of TCGA transcriptomes^70^). The transcripts per million (TPM) abundance have been compared and found coherent with the median TPM of the 100 most expressed genes in lung tissues reported in the Genotype-Tissue Expression (GTEx) (https://gtexportal.org/home/eqtls/tissue?tissueName=Lung).

### TCGA data

TCGA tumour and normal raw counts data have been downloaded from the Genomic Data Common (GDC) portal (https://portal.gdc.cancer.gov/legacy-archive/search/f), selecting all the samples related to TCGA-LUAD and TCGA-LUSC projects from the Pan-Cancer Atlas cohort. Following the same procedure adopted for PROMOLE samples, we filtered the subset of the protein coding genes common to the two datasets and transformed gene expression data into TPM, using the gene lengths reported in the GRCh38.p12 version of the reference genome. TCGA data also includes 33 cases of stage IV NSCLC.

### Data integration

To ensure integrability between PROMOLE and TCGA gene expression datasets, a batch-effect estimation has been performed via principal variance component analysis (PVCA)^71^. The percentage of the total variance which has been imputed to the study (PROMOLE vs TCGA) is 2.7%, which can be considered negligible (see Supplementary Fig. 3).

### Consensus clustering

We implemented an ensemble learning model based on consensus clustering applied to log2-scaled gene expression data. The method is deployed in two phases (see Fig. 2a):

1. different clustering methods are run independently on the same data and the resulting cluster associations are obtained for each patient and method;
2. clustering associations are compared and a voting process is established, in order to detect the most supported label for each patient.

#### Phase1

In our study, the ML clustering methods employed for consensus clustering cover most of the state-of-the-art and well-established algorithms. Specifically, we employed K-Means clustering^72^, spectral clustering^73^, hierarchical clustering^74^, iClusterPlus^14^, and SNF^75^, the first three methods being of general applicability, whereas the last two have been specifically designed for genomic data analysis. The main rationale was to employ a wide variety of models and approaches, in order to maximise robustness. It has to be noted that K-Means, Hierarchical and Spectral clustering have been considered in two versions: a first one where the clustering technique is adopted directly on the joint TCGA-PROMOLE dataset, and a second one where the dataset has been previously treated with a Self-Organizing Map (SOM) or Kohonen’s Map^76,77^ before clustering. In fact, SOMs are used to produce a low-dimensional representation of a higher dimensional dataset still preserving its topological structure and already proved to be effective on gene-expression data^78^, in combination with K-Means, hierarchical and consensus clustering, for different types of tumour subtyping tasks^79,80^. SOMs were not applied on iClusterPlus and SNF since these methods were already designed to deal with high-dimensional genomic matrices.

Each method was employed with three different seeds, in order for it to be tested with three different initialization points. Moreover, four maximum number of clusters were employed (3, 4, 5, and 6 clusters, see Fig. 1 and Supplementary Fig. 4 and 7) in order to identify the most suitable number of classes.

#### Phase2

The results of Phase1 are used as input of the subsequent voting process (see Fig. 2a). Since clustering methods are completely unsupervised and do not rely on any sort of pre-processed label, one of the difficulties of consensus clustering is normalizing the nomenclature of all the different clustering methods to make them comparable. Among the models tested in Phase1, we defined a reference model, as the one with the best separation between LUAD and LUSC. The reference method is used in order to “translate” all the other clustering methods in a common nomenclature employing the mode operation: the classes of the tested methods are associated to the ones of the reference by computing the most frequently voted tested-class among each reference ones (see Fig. 2b). After all the clustering methods have been correctly normalized to the same nomenclature, the final voting is performed by considering the most frequently voted class for each patient (see exemplary Supplementary Fig. 8).

### Downstream analysis

The four groups have been compared on different levels. A first characterization can be seen in how clustering patients reflects on their clinical features and on the disease-free survival. Moreover, the biological pathway corresponding to each expression profile as well as the mutational landscape of the identified subgroups has been explored.

#### Clinical features

The distributions of the clinical variables of interest (age, gender, stage, smoking history, histological type) have been compared among the four clusters. The relative abundances of such variables for each emerging cluster have been tested using χ^2^ (see Fig. 3) test and Z-tests^81^ against their respective null models.

Null models have been built via bootstrap resampling of the consensus clustering labels and provide the statistically expected abundance of each variable in each cluster, if no correlation is assumed, given the distribution of the variable itself over the whole dataset and the numerosity of samples in each cluster. In particular, the consensus labels have been resampled 1000 times, finally computing the average abundance of each variable for each cluster and its standard deviation. The Z-score is built as the difference between observed and relative abundance in units of standard deviation.

#### Survival analysis

The DFS^82^ was defined as the time from the initial treatment to recurrence of tumor. The null hypothesis that there is no difference among clusters in the probability of cancer recurrence has been tested with a log-rank test^83^.

A Cox regression model^84^ was fitted to identify significant regressors among clinical features and clustering. The following clinical variables have been tested: histological subtype (LUSC or LUAD), age at diagnosis (< 65, 65+), sex, tumour stage, smoking history (lifelong non-smoker, current smoker or current reformed smoker) and clustering. Only the significant regressors have been tested in the final model to calculate the Hazard Ratios (HR). Cox models, HR and Kaplan–Meier curves have been computed and plotted using the R survival^85^ and survminer^86^ packages. Please note that, since information about treatment received was missing for the vast majority of the patients (~ 70%), we did not distinguish between groups receiving different treatments. However, we observed that the percentage of patients receiving the same treatment was approximately uniform across the four clusters.

#### Functional annotation

Functional annotation of the four main transcriptional clusters was based on the GSEA^87^ desktop application from the BROAD Institute (https://software.broadinstitute.org/cancer/software/gsea/wiki/index.php/Main_Page), with standard parameters. The gene set databases “GeneOntology–BiologicalProcesses”^75^, “KEGG pathways” and “HALLMARK of Cancer” from the Molecular Signatures Database^34^ (MSigDB, http://www.gsea-msigdb.org/gsea/msigdb/collections.jsp) were used as reference for the annotation. In all the cases, we loaded the entire RNA-seq dataset in the GSEA application and we performed a comparison between each of the four clusters versus the remaining samples. Only results associated with an FDR q-value < 0.1 were retained for further analysis. Each macro-category was reported in Fig. 5, according the positive/negative NES values and labelled with the FDR value plus the number of associated genes corresponding to the larger gene set in terms of genes included in the macro-category. To this end, we defined a set of 13, manually curated, major functional macro-categories (see Supplementary Materials), which allowed us to group and better summarize related functional classes found over-represented.

#### Exome: over-/under-representation analysis

We performed an over-representation analysis of the mutations characterising each subgroup by testing the observed number of mutations for each gene and each emerging cluster with Z-tests against their respective null models. Null models have been built following the same procedure described in the case of clinical features.

### Ethics approval and consent to participate

The prospective observational clinical trial PROMOLE has been approved by the Ethical Committee of the hospital AOU San Luigi Gonzaga (Orbassano, I) (protocol n.14057 approved in September 28, 2018, n.73/2018). Written informed consent has been prospectively collected for each participant.

## Supplementary Information

Supplementary Information 1.

Supplementary Information 2.

## Data Availability

The results published here are predominantly based (889 patients) upon data generated by the TCGA Research Network (https://www.cancer.gov/tcga) and available in the Genomic Data Commons (GDC) repository (see “Methods”). The genetic and clinical data generated as part of the DEFLeCT project (81 patients) has been deposited at the European Genome-phenome Archive (EGA), which is hosted by the EBI and the CRG, under accession number EGAS00001007219 (metadata are available under accession number EGAD00001010838).

## Code availability

The code implementing our consensus clustering approach is publicly available on GitLab at: https://gitlab.com/deflect-public/consensus-clustering.
