# Supplementary material for: Consensus clustering methodology to improve molecular stratification of non-small cell lung cancer
Source: Sci Rep. 2023 May 12;13:7759. doi: 10.1038/s41598-023-33954-x (PMC10182023; doi:10.1038/s41598-023-33954-x)
Supplement: Supplementary file 1 — Supplementary Information 1. [file 41598_2023_33954_MOESM1_ESM.pdf]

# MSigDB annotations - CELL PROLIFERATION

GOBP_REGULATION_OF_EXTENT_OF_CELL_GROWTH
GOBP_REGULATION_OF_MITOTIC_CELL_CYCLE
GOBP_REGULATION_OF_MITOTIC_CELL_CYCLE_PHASE_TRANSITION
GOBP_REGULATION_OF_MITOTIC_NUCLEAR_DIVISION
GOBP_REGULATION_OF_MITOTIC_SISTER_CHROMATID_SEGREGATION
GOBP_REGULATION_OF_NUCLEAR_DIVISION
GOBP_REGULATION_OF_NUCLEOCYTOPLASMIC_TRANSPORT
GOBP_REGULATION_OF_TRANSCRIPTION_INVOLVED_IN_G1_S_TRANSITION_OF_MITOTIC_CELL_CYCLE
GOBP_REPLICATION_FORK_PROCESSING
GOBP_REPLICATIVE_SENESCENCE
GOBP_ERBB_SIGNALING_PATHWAY
GOBP_NEGATIVE_REGULATION_OF_ERBB_SIGNALING_PATHWAY
GOBP_NEGATIVE_REGULATION_OF_ANOIKIS
GOBP_REGULATION_OF_CYTOKINESIS
GOBP_REGULATION_OF_SIGNAL_TRANSDUCTION_BY_P53_CLASS_MEDIATOR
KEGG_APOPTOSIS
GOBP_AGING
GOBP_ANAPHASE_PROMOTING_COMPLEX_DEPENDENT_CATABOLIC_PROCESS
GOBP_ATTACHMENT_OF_SPINDLE_MICROTUBULES_TO_KINETOCHORE
GOBP_BASE_CONVERSION_OR_SUBSTITUTION_EDITING
GOBP_CELL_CYCLE_DNA_REPLICATION
GOBP_CELL_CYCLE_G1_S_PHASE_TRANSITION
GOBP_CELL_CYCLE_G2_M_PHASE_TRANSITION
GOBP_CELL_CYCLE_PHASE_TRANSITION
GOBP_CENTRIOLE_ASSEMBLY
GOBP_CENTROMERE_COMPLEX_ASSEMBLY
GOBP_CHROMATIN_ASSEMBLY_OR_DISASSEMBLY
GOBP_CHROMATIN_REMODELING
GOBP_CHROMOSOME_CONDENSATION
GOBP_CHROMOSOME_LOCALIZATION
GOBP_CHROMOSOME_ORGANIZATION_INVOLVED_IN_MEIOTIC_CELL_CYCLE
GOBP_CHROMOSOME_SEGREGATION
GOBP_CHROMOSOME_SEPARATION
GOBP_MEIOSIS_I_CELL_CYCLE_PROCESS
GOBP_MEIOTIC_CELL_CYCLE
GOBP_MEIOTIC_CELL_CYCLE_PROCESS
GOBP_MEIOTIC_CHROMOSOME_SEGREGATION
GOBP_METAPHASE_PLATE_CONGRESSION
GOBP_MICROTUBULE_ANCHORING
GOBP_MICROTUBULE_CYTOSKELETON_ORGANIZATION_INVOLVED_IN_MITOSIS
GOBP_MICROTUBULE_DEPOLYMERIZATION
GOBP_MICROTUBULE_ORGANIZING_CENTER_ORGANIZATION
GOBP_MITOTIC_CELL_CYCLE_PHASE_TRANSITION
GOBP_MITOTIC_CHROMOSOME_CONDENSATION
GOBP_MITOTIC_NUCLEAR_DIVISION
GOBP_MITOTIC_SISTER_CHROMATID_COHESION
GOBP_MITOTIC_SISTER_CHROMATID_SEGREGATION
GOBP_MITOTIC_SPINDLE_ASSEMBLY
GOBP_MITOTIC_SPINDLE_ORGANIZATION
GOBP_NECROTIC_CELL_DEATH
GOBP_NEGATIVE_REGULATION_OF_CELL_CYCLE_G1_S_PHASE_TRANSITION
GOBP_NEGATIVE_REGULATION_OF_CELL_CYCLE_PHASE_TRANSITION

GOBP_NEGATIVE_REGULATION_OF_CELL_GROWTH
GOBP_NEGATIVE_REGULATION_OF_CHROMOSOME_ORGANIZATION
GOBP_NEGATIVE_REGULATION_OF_GROWTH
GOBP_NEGATIVE_REGULATION_OF_METAPHASE_ANAPHASE_TRANSITION_OF_CELL_CYCLE
GOBP_NEGATIVE_REGULATION_OF_MITOTIC_CELL_CYCLE
GOBP_NEGATIVE_REGULATION_OF_MITOTIC_CELL_CYCLE_PHASE_TRANSITION
GOBP_POSITIVE_REGULATION_OF_CELL_CYCLE
GOBP_POSITIVE_REGULATION_OF_CELL_CYCLE_G2_M_PHASE_TRANSITION
GOBP_POSITIVE_REGULATION_OF_CELL_CYCLE_PHASE_TRANSITION
GOBP_POSITIVE_REGULATION_OF_CELL_CYCLE_PROCESS
GOBP_POSITIVE_REGULATION_OF_CELL_DIVISION
GOBP_POSITIVE_REGULATION_OF_CYCLIN_DEPENDENT_PROTEIN_KINASE_ACTIVITY
GOBP_POSITIVE_REGULATION_OF_MEIOTIC_NUCLEAR_DIVISION
GOBP_POSITIVE_REGULATION_OF_MITOTIC_CELL_CYCLE
GOBP_POSITIVE_REGULATION_OF_MITOTIC_CELL_CYCLE_PHASE_TRANSITION
GOBP_REGULATION_OF_CELL_CYCLE_G1_S_PHASE_TRANSITION
GOBP_REGULATION_OF_CELL_CYCLE_G2_M_PHASE_TRANSITION
GOBP_REGULATION_OF_CELL_CYCLE_PHASE_TRANSITION
GOBP_REGULATION_OF_CELL_DIVISION
GOBP_SIGNAL_TRANSDUCTION_BY_P53_CLASS_MEDIATOR
HALLMARK_E2F_TARGETS
HALLMARK_G2M_CHECKPOINT
HALLMARK_MITOTIC_SPINDLE
HALLMARK_MTORC1_SIGNALING
HALLMARK_MYC_TARGETS_V1
HALLMARK_MYC_TARGETS_V2
HALLMARK_P53_PATHWAY
KEGG_CELL_CYCLE
KEGG_ERBB_SIGNALING_PATHWAY
KEGG_P53_SIGNALING_PATHWAY

# MSigDB annotations - CYTOSKELETON
GOBP_ACTIN_CYTOSKELETON_REORGANIZATION
GOBP_ACTIN_FILAMENT_BASED_MOVEMENT
GOBP_ACTIN_FILAMENT_BASED_TRANSPORT
GOBP_ACTIN_FILAMENT_BUNDLE_ORGANIZATION
GOBP_ACTIN_FILAMENT_DEPOLYMERIZATION
GOBP_ACTIN_FILAMENT_ORGANIZATION
GOBP_ACTIN_FILAMENT_POLYMERIZATION
GOBP_ACTIN_NUCLEATION
GOBP_ACTIN_POLYMERIZATION_OR_DEPOLYMERIZATION
GOBP_AMEBOIDAL_TYPE_CELL_MIGRATION
GOBP_NEGATIVE_REGULATION_OF_ACTIN_FILAMENT_BUNDLE_ASSEMBLY
GOBP_NEGATIVE_REGULATION_OF_CYTOSKELETON_ORGANIZATION
GOBP_POSITIVE_REGULATION_OF_ACTIN_CYTOSKELETON_REORGANIZATION
GOBP_POSITIVE_REGULATION_OF_ACTIN_FILAMENT_BUNDLE_ASSEMBLY
GOBP_POSITIVE_REGULATION_OF_ACTIN_FILAMENT_POLYMERIZATION
GOBP_POSITIVE_REGULATION_OF_ACTIN_NUCLEATION
GOBP_POSITIVE_REGULATION_OF_STRESS_FIBER_ASSEMBLY
GOBP_POSITIVE_REGULATION_OF_SUPRAMOLECULAR_FIBER_ORGANIZATION
GOBP_REGULATION_OF_ACTIN_CYTOSKELETON_REORGANIZATION
GOBP_REGULATION_OF_ACTIN_FILAMENT_BASED_PROCESS

GOBP_REGULATION_OF_ACTIN_FILAMENT_BUNDLE_ASSEMBLY
GOBP_REGULATION_OF_ACTIN_FILAMENT_LENGTH
GOBP_REGULATION_OF_ACTIN_FILAMENT_ORGANIZATION
GOBP_REGULATION_OF_ACTOMYOSIN_STRUCTURE_ORGANIZATION
GOBP_POSITIVE_REGULATION_OF_CYTOSKELETON_ORGANIZATION
GOBP_REGULATION_OF_CYTOSKELETON_ORGANIZATION
GOBP_POSITIVE_REGULATION_OF_ACTIN_CYTOSKELETON_REORGANIZATION
GOBP_POSITIVE_REGULATION_OF_ACTIN_FILAMENT_BUNDLE_ASSEMBLY
GOBP_POSITIVE_REGULATION_OF_ACTIN_FILAMENT_POLYMERIZATION
GOBP_POSITIVE_REGULATION_OF_ACTIN_NUCLEATION
KEGG_REGULATION_OF_ACTIN_CYTOSKELETON

# MSigDB annotations - DNA and RNA PROCESSING
GOBP_CLEAVAGE_INVOLVED_IN_RRNA_PROCESSING
GOBP_DNA_BIOSYNTHETIC_PROCESS
GOBP_DNA_CONFORMATION_CHANGE
GOBP_DNA_DEALKYLATION
GOBP_DNA_DEPENDENT_DNA_REPLICATION
GOBP_DNA_DEPENDENT_DNA_REPLICATION_MAINTENANCE_OF_FIDELITY
GOBP_DNA_DOUBLE_STRAND_BREAK_PROCESSING
GOBP_DNA_GEOMETRIC_CHANGE
GOBP_DNA_METHYLATION_DEPENDENT_HETEROCHROMATIN_ASSEMBLY
GOBP_DNA_METHYLATION_OR_DEMETHYLATION
GOBP_DNA_RECOMBINATION
GOBP_DNA_REPAIR
GOBP_DNA_REPLICATION
GOBP_DNA_REPLICATION_CHECKPOINT_SIGNALING
GOBP_DNA_REPLICATION_INDEPENDENT_CHROMATIN_ORGANIZATION
GOBP_DNA_REPLICATION_INITIATION
GOBP_DNA_STRAND_ELONGATION
GOBP_DNA_STRAND_ELONGATION_INVOLVED_IN_DNA_REPLICATION
GOBP_DNA_UNWINDING_INVOLVED_IN_DNA_REPLICATION
GOBP_DOUBLE_STRAND_BREAK_REPAIR
GOBP_DOUBLE_STRAND_BREAK_REPAIR_VIA_NONHOMOLOGOUS_END_JOINING
GOBP_ESTABLISHMENT_OF_PROTEIN_LOCALIZATION_TO_CHROMOSOME
GOBP_ESTABLISHMENT_OF_PROTEIN_LOCALIZATION_TO_TELOMERE
GOBP_ESTABLISHMENT_OF_SPINDLE_ORIENTATION
GOBP_FACULTATIVE_HETEROCHROMATIN_ASSEMBLY
GOBP_HETEROCHROMATIN_ORGANIZATION
GOBP_HISTONE_EXCHANGE
GOBP_HOMOLOGOUS_RECOMBINATION
GOBP_INTERSTRAND_CROSS_LINK_REPAIR
GOBP_INTRINSIC_APOPTOTIC_SIGNALING_PATHWAY_IN_RESPONSE_TO_DNA_DAMAGE
GOBP_KINETOCHORE_ORGANIZATION
GOBP_MATURATION_OF_5_8S_RRNA
GOBP_MATURATION_OF_5_8S_RRNA_FROM_TRICISTRONIC_RRNA_TRANSCRIPT_SSU_RRNA_5_8S_RRNA_LSU_RRNA
GOBP_MATURATION_OF_SSU_RRNA
GOBP_MATURATION_OF_SSU_RRNA_FROM_TRICISTRONIC_RRNA_TRANSCRIPT_SSU_RRNA_5_8S_RRNA_LSU_RRNA
GOBP_MRNA_MODIFICATION
GOBP_MRNA_TRANSPORT
GOBP_NEGATIVE_REGULATION_OF_DNA_BINDING
GOBP_NEGATIVE_REGULATION_OF_DNA_BINDING_TRANSCRIPTION_FACTOR_ACTIVITY

GOBP_NEGATIVE_REGULATION_OF_DNA_METABOLIC_PROCESS
GOBP_NEGATIVE_REGULATION_OF_DNA_REPLICATION
GOBP_NEGATIVE_REGULATION_OF_GENE_EXPRESSION_EPIGENETIC
GOBP_NEGATIVE_REGULATION_OF_HISTONE_MODIFICATION
GOBP_NUCLEAR_CHROMOSOME_SEGREGATION
GOBP_NUCLEAR_EXPORT
GOBP_NUCLEAR_MIGRATION
GOBP_NUCLEAR_TRANSPORT
GOBP_NUCLEOBASE_BIOSYNTHETIC_PROCESS
GOBP_NUCLEOBASE_CONTAINING_SMALL_MOLECULE_CATABOLIC_PROCESS
GOBP_NUCLEOSIDE_CATABOLIC_PROCESS
GOBP_NUCLEOSIDE_MONOPHOSPHATE_BIOSYNTHETIC_PROCESS
GOBP_NUCLEOSOME_ASSEMBLY
GOBP_NUCLEOSOME_ORGANIZATION
GOBP_NUCLEOTIDE_BINDING_DOMAIN_LEUCINE_RICH_REPEAT_CONTAINING_RECEPTOR_SIGNALING_PATHWAY
GOBP_NUCLEOTIDE_SALVAGE
GOBP_NUCLEOTIDE_TRANSMEMBRANE_TRANSPORT
GOBP_NUCLEOTIDE_TRANSPORT
GOBP_NUCLEUS_LOCALIZATION
GOBP_POSITIVE_REGULATION_OF_DNA_BINDING_TRANSCRIPTION_FACTOR_ACTIVITY
GOBP_POSITIVE_REGULATION_OF_DNA_BIOSYNTHETIC_PROCESS
GOBP_POSITIVE_REGULATION_OF_DNA_METABOLIC_PROCESS
GOBP_POSITIVE_REGULATION_OF_DNA_REPAIR
GOBP_POSITIVE_REGULATION_OF_RESPONSE_TO_DNA_DAMAGE_STIMULUS
GOBP_POSITIVE_REGULATION_OF_TELOMERASE_ACTIVITY
GOBP_POSITIVE_REGULATION_OF_TELOMERASE_RNA_LOCALIZATION_TO_CAJAL_BODY
GOBP_POSITIVE_REGULATION_OF_TELOMERE_MAINTENANCE
GOBP_POSITIVE_REGULATION_OF_TELOMERE_MAINTENANCE_VIA_TELOMERE_LENGTHENING
GOBP_POSITIVE_REGULATION_OF_TRANSCRIPTION_BY_RNA_POLYMERASE_I
GOBP_PROTEIN_DNA_COMPLEX_ASSEMBLY
GOBP_PROTEIN_DNA_COMPLEX_SUBUNIT_ORGANIZATION
GOBP_PURINERGIC_NUCLEOTIDE_RECEPTOR_SIGNALING_PATHWAY
GOBP_PURINE_NUCLEOSIDE_MONOPHOSPHATE_BIOSYNTHETIC_PROCESS
GOBP_PYRIMIDINE_NUCLEOSIDE_CATABOLIC_PROCESS
GOBP_PYRIMIDINE_NUCLEOSIDE_TRIPHOSPHATE_METABOLIC_PROCESS
GOBP_RECOMBINATIONAL_REPAIR
GOBP_REGULATION_OF_CENTRIOLE_REPLICATION
GOBP_REGULATION_OF_CHROMOSOME_ORGANIZATION
GOBP_REGULATION_OF_CHROMOSOME_SEGREGATION
GOBP_REGULATION_OF_CHROMOSOME_SEPARATION
GOBP_REGULATION_OF_DNA_BINDING
GOBP_REGULATION_OF_DNA_BINDING_TRANSCRIPTION_FACTOR_ACTIVITY
GOBP_REGULATION_OF_DNA_BIOSYNTHETIC_PROCESS
GOBP_REGULATION_OF_DNA_DAMAGE_RESPONSE_SIGNAL_TRANSDUCTION_BY_P53_CLASS_MEDIATOR
GOBP_REGULATION_OF_DNA_DEPENDENT_DNA_REPLICATION
GOBP_REGULATION_OF_DNA_METABOLIC_PROCESS
GOBP_REGULATION_OF_DNA_METHYLATION
GOBP_REGULATION_OF_DNA_METHYLATION_DEPENDENT_HETEROCHROMATIN_ASSEMBLY
GOBP_REGULATION_OF_DNA_REPAIR
GOBP_REGULATION_OF_DNA_REPLICATION
GOBP_REGULATION_OF_DOUBLE_STRAND_BREAK_REPAIR
GOBP_REGULATION_OF_DOUBLE_STRAND_BREAK_REPAIR_VIA_HOMOLOGOUS_RECOMBINATION
GOBP_REGULATION_OF_HISTONE_H3_K9_METHYLATION

GOBP_REGULATION_OF_RESPONSE_TO_DNA_DAMAGE_STIMULUS
GOBP_REGULATION_OF_TELOMERE_MAINTENANCE
GOBP_REGULATION_OF_TELOMERE_MAINTENANCE_VIA_TELOMERE_LENGTHENING
GOBP_REGULATION_OF_TRANSCRIPTION_BY_RNA_POLYMERASE_I
GOBP_RIBONUCLEOPROTEIN_COMPLEX_BIOGENESIS
GOBP_RIBONUCLEOPROTEIN_COMPLEX_SUBUNIT_ORGANIZATION
GOBP_RIBONUCLEOSIDE_MONOPHOSPHATE_BIOSYNTHETIC_PROCESS
GOBP_RIBOSOMAL_SMALL_SUBUNIT_BIOGENESIS
GOBP_RIBOSOME_ASSEMBLY
GOBP_RIBOSOME_BIOGENESIS
GOBP_RNA_5_END_PROCESSING
GOBP_RNA_DEPENDENT_DNA_BIOSYNTHETIC_PROCESS
GOBP_RNA_EXPORT_FROM_NUCLEUS
GOBP_RRNA_METABOLIC_PROCESS
GOBP_RRNA_MODIFICATION
GOBP_RRNA_TRANSCRIPTION
GOBP_SIGNAL_TRANSDUCTION_IN_RESPONSE_TO_DNA_DAMAGE
GOBP_SINGLE_STRANDED_VIRAL_RNA_REPLICATION_VIA_DOUBLE_STRANDED_DNA_INTERMEDIATE
GOBP_SISTER_CHROMATID_COHESION
GOBP_SISTER_CHROMATID_SEGREGATION
GOBP_SPINDLE_ASSEMBLY
GOBP_SPINDLE_LOCALIZATION
GOBP_SPINDLE_ORGANIZATION
GOBP_SPLICEOSOMAL_SNRNP_ASSEMBLY
GOBP_TELOMERASE_RNA_LOCALIZATION
GOBP_TELOMERE_MAINTENANCE
GOBP_TELOMERE_MAINTENANCE_VIA_TELOMERE_LENGTHENING
GOBP_TELOMERE_ORGANIZATION
GOBP_TRANSCRIPTION_BY_RNA_POLYMERASE_I
GOBP_REGULATION_OF_SISTER_CHROMATID_COHESION
KEGG_DNA_REPLICATION
KEGG_HOMOLOGOUS_RECOMBINATION
KEGG_MISMATCH_REPAIR
KEGG_NUCLEOTIDE_EXCISION_REPAIR
KEGG_RNA_POLYMERASE
KEGG_SPLICEOSOME

# MSigDB annotations - EPITHELIUM
GOBP_EPIDERMAL_CELL_DIFFERENTIATION
GOBP_EPITHELIAL_CELL_APOPTOTIC_PROCESS
GOBP_EPITHELIAL_CELL_DEVELOPMENT
GOBP_EPITHELIAL_CELL_PROLIFERATION
GOBP_EPITHELIAL_TO_MESENCHYMAL_TRANSITION
GOBP_ESTABLISHMENT_OF_CELL_POLARITY
GOBP_ESTABLISHMENT_OF_EPITHELIAL_CELL_APICAL_BASAL_POLARITY
GOBP_ESTABLISHMENT_OF_EPITHELIAL_CELL_POLARITY
GOBP_KERATINIZATION
GOBP_MESENCHYMAL_CELL_DIFFERENTIATION
GOBP_MESENCHYMAL_CELL_PROLIFERATION
GOBP_MORPHOGENESIS_OF_AN_EPITHELIAL_BUD
GOBP_MORPHOGENESIS_OF_AN_EPITHELIAL_SHEET
GOBP_MORPHOGENESIS_OF_AN_EPITHELIUM

GOBP_MORPHOGENESIS_OF_A_POLARIZED_EPITHELIUM
GOBP_NEGATIVE_REGULATION_OF_EPITHELIAL_CELL_DIFFERENTIATION
GOBP_NEGATIVE_REGULATION_OF_EPITHELIAL_CELL_MIGRATION
GOBP_NEGATIVE_REGULATION_OF_EPITHELIAL_CELL_PROLIFERATION
GOBP_POLARIZED_EPITHELIAL_CELL_DIFFERENTIATION
GOBP_POSITIVE_REGULATION_OF_EPITHELIAL_CELL_APOPTOTIC_PROCESS
GOBP_POSITIVE_REGULATION_OF_EPITHELIAL_CELL_MIGRATION
GOBP_POSITIVE_REGULATION_OF_EPITHELIAL_CELL_PROLIFERATION
GOBP_POSITIVE_REGULATION_OF_EPITHELIAL_TO_MESENCHYMAL_TRANSITION
GOBP_REGULATION_OF_EPITHELIAL_CELL_MIGRATION
GOBP_REGULATION_OF_EPITHELIAL_TO_MESENCHYMAL_TRANSITION
GOBP_REGULATION_OF_KERATINOCYTE_PROLIFERATION
GOBP_REGULATION_OF_MESENCHYMAL_CELL_PROLIFERATION

# MSigDB annotations - IMMUNE SYSTEM
GOBP_ACTIVATED_T_CELL_PROLIFERATION
GOBP_ACTIVATION_OF_IMMUNE_RESPONSE
GOBP_ADAPTIVE_IMMUNE_RESPONSE
GOBP_ADAPTIVE_IMMUNE_RESPONSE_BASED_ON_SOMATIC_RECOMBINATION_OF_IMMUNE_RECEPTORS_BUILT_FR
GOBP_ALPHA_BETA_T_CELL_ACTIVATION
GOBP_ALPHA_BETA_T_CELL_DIFFERENTIATION
GOBP_ALPHA_BETA_T_CELL_PROLIFERATION
GOBP_ANTIGEN_PROCESSING_AND_PRESENTATION
GOBP_ANTIGEN_PROCESSING_AND_PRESENTATION_OF_EXOGENOUS_ANTIGEN
GOBP_ANTIGEN_PROCESSING_AND_PRESENTATION_OF_EXOGENOUS_PEPTIDE_ANTIGEN
GOBP_ANTIGEN_PROCESSING_AND_PRESENTATION_OF_PEPTIDE_ANTIGEN
GOBP_ANTIGEN_PROCESSING_AND_PRESENTATION_OF_PEPTIDE_OR_POLYSACCHARIDE_ANTIGEN_VIA_MHC_CLASS_
GOBP_ANTIGEN_RECEPTOR_MEDIATED_SIGNALING_PATHWAY
GOBP_BIOLOGICAL_PROCESS_INVOLVED_IN_INTERACTION_WITH_HOST
GOBP_BIOLOGICAL_PROCESS_INVOLVED_IN_SYMBIOTIC_INTERACTION
GOBP_B_CELL_ACTIVATION
GOBP_B_CELL_ACTIVATION_INVOLVED_IN_IMMUNE_RESPONSE
GOBP_B_CELL_APOPTOTIC_PROCESS
GOBP_B_CELL_DIFFERENTIATION
GOBP_B_CELL_HOMEOSTASIS
GOBP_B_CELL_MEDIATED_IMMUNITY
GOBP_B_CELL_PROLIFERATION
GOBP_B_CELL_RECEPTOR_SIGNALING_PATHWAY
GOBP_CD4_POSITIVE_ALPHA_BETA_T_CELL_ACTIVATION
GOBP_CD4_POSITIVE_ALPHA_BETA_T_CELL_DIFFERENTIATION
GOBP_CD4_POSITIVE_OR_CD8_POSITIVE_ALPHA_BETA_T_CELL_LINEAGE_COMMITMENT
GOBP_CD8_POSITIVE_ALPHA_BETA_T_CELL_ACTIVATION
GOBP_CELLULAR_RESPONSE_TO_ABIOTIC_STIMULUS
GOBP_CELLULAR_RESPONSE_TO_ACID_CHEMICAL
GOBP_CELLULAR_RESPONSE_TO_AMYLOID_BETA
GOBP_CELL_ACTIVATION_INVOLVED_IN_IMMUNE_RESPONSE
GOBP_COMPLEMENT_ACTIVATION
GOBP_CYTOKINE_PRODUCTION_INVOLVED_IN_IMMUNE_RESPONSE
GOBP_DEFENSE_RESPONSE_TO_BACTERIUM
GOBP_DEFENSE_RESPONSE_TO_GRAM_POSITIVE_BACTERIUM
GOBP_DEFENSE_RESPONSE_TO_SYMBIONT
GOBP_FC_EPSILON_RECEPTOR_SIGNALING_PATHWAY

GOBP_FC_GAMMA_RECEPTOR_SIGNALING_PATHWAY
GOBP_FC_RECEPTOR_MEDIATED_STIMULATORY_SIGNALING_PATHWAY
GOBP_FC_RECEPTOR_SIGNALING_PATHWAY
GOBP_GAMMA_DELTA_T_CELL_ACTIVATION
GOBP_GRANULOCYTE_ACTIVATION
GOBP_GRANULOCYTE_CHEMOTAXIS
GOBP_GRANULOCYTE_DIFFERENTIATION
GOBP_GRANULOCYTE_MIGRATION
GOBP_HUMORAL_IMMUNE_RESPONSE_MEDIATED_BY_CIRCULATING_IMMUNOGLOBULIN
GOBP_IMMUNE_RESPONSE_REGULATING_CELL_SURFACE_RECEPTOR_SIGNALING_PATHWAY
GOBP_IMMUNE_RESPONSE_REGULATING_SIGNALING_PATHWAY
GOBP_IMMUNE_RESPONSE_TO_TUMOR_CELL
GOBP_IMMUNOGLOBULIN_PRODUCTION_INVOLVED_IN_IMMUNOGLOBULIN_MEDIATED_IMMUNE_RESPONSE
GOBP_IMMUNOLOGICAL_SYNAPSE_FORMATION
GOBP_LEUKOCYTE_ADHESION_TO_VASCULAR_ENDOTHELIAL_CELL
GOBP_LEUKOCYTE_APOPTOTIC_PROCESS
GOBP_LEUKOCYTE_CELL_CELL_ADHESION
GOBP_LEUKOCYTE_CHEMOTAXIS
GOBP_LEUKOCYTE_DEGRANULATION
GOBP_LEUKOCYTE_HOMEOSTASIS
GOBP_LEUKOCYTE_MEDIATED_CYTOTOXICITY
GOBP_LEUKOCYTE_MEDIATED_IMMUNITY
GOBP_LEUKOCYTE_MIGRATION
GOBP_LEUKOCYTE_PROLIFERATION
GOBP_LEUKOCYTE_TETHERING_OR_ROLLING
GOBP_LYMPHOCYTE_ACTIVATION_INVOLVED_IN_IMMUNE_RESPONSE
GOBP_LYMPHOCYTE_APOPTOTIC_PROCESS
GOBP_LYMPHOCYTE_COSTIMULATION
GOBP_LYMPHOCYTE_HOMEOSTASIS
GOBP_LYMPHOCYTE_MEDIATED_IMMUNITY
GOBP_LYMPHOCYTE_MIGRATION
GOBP_MACROPHAGE_ACTIVATION
GOBP_MACROPHAGE_ACTIVATION_INVOLVED_IN_IMMUNE_RESPONSE
GOBP_MACROPHAGE_CHEMOTAXIS
GOBP_MACROPHAGE_CYTOKINE_PRODUCTION
GOBP_MACROPHAGE_DIFFERENTIATION
GOBP_MACROPHAGE_MIGRATION
GOBP_MEGAKARYOCYTE_DEVELOPMENT
GOBP_MEGAKARYOCYTE_DIFFERENTIATION
GOBP_MHC_CLASS_II_BIOSYNTHETIC_PROCESS
GOBP_MONOCYTE_CHEMOTACTIC_PROTEIN_1_PRODUCTION
GOBP_MONOCYTE_CHEMOTAXIS
GOBP_MONOCYTE_DIFFERENTIATION
GOBP_MONONUCLEAR_CELL_DIFFERENTIATION
GOBP_MONONUCLEAR_CELL_MIGRATION
GOBP_MYD88_DEPENDENT_TOLL_LIKE_RECEPTOR_SIGNALING_PATHWAY
GOBP_MYELOID_CELL_ACTIVATION_INVOLVED_IN_IMMUNE_RESPONSE
GOBP_MYELOID_CELL_APOPTOTIC_PROCESS
GOBP_MYELOID_CELL_DEVELOPMENT
GOBP_MYELOID_CELL_DIFFERENTIATION
GOBP_MYELOID_CELL_HOMEOSTASIS
GOBP_MYELOID_DENDRITIC_CELL_ACTIVATION
GOBP_MYELOID_LEUKOCYTE_ACTIVATION

GOBP_MYELOID_LEUKOCYTE_CYTOKINE_PRODUCTION
GOBP_MYELOID_LEUKOCYTE_DIFFERENTIATION
GOBP_MYELOID_LEUKOCYTE_MEDIATED_IMMUNITY
GOBP_MYELOID_LEUKOCYTE_MIGRATION
GOBP_NATURAL_KILLER_CELL_ACTIVATION
GOBP_NATURAL_KILLER_CELL_ACTIVATION_INVOLVED_IN_IMMUNE_RESPONSE
GOBP_NATURAL_KILLER_CELL_DIFFERENTIATION
GOBP_NATURAL_KILLER_CELL_MEDIATED_IMMUNITY
GOBP_NEGATIVE_REGULATION_OF_ADAPTIVE_IMMUNE_RESPONSE
GOBP_NEGATIVE_REGULATION_OF_ALPHA_BETA_T_CELL_ACTIVATION
GOBP_NEGATIVE_REGULATION_OF_ALPHA_BETA_T_CELL_DIFFERENTIATION
GOBP_NEGATIVE_REGULATION_OF_B_CELL_ACTIVATION
GOBP_NEGATIVE_REGULATION_OF_B_CELL_MEDIATED_IMMUNITY
GOBP_NEGATIVE_REGULATION_OF_B_CELL_PROLIFERATION
GOBP_NEGATIVE_REGULATION_OF_CD4_POSITIVE_ALPHA_BETA_T_CELL_ACTIVATION
GOBP_NEGATIVE_REGULATION_OF_CD4_POSITIVE_ALPHA_BETA_T_CELL_DIFFERENTIATION
GOBP_NEGATIVE_REGULATION_OF_DEFENSE_RESPONSE
GOBP_NEGATIVE_REGULATION_OF_HUMORAL_IMMUNE_RESPONSE
GOBP_NEGATIVE_REGULATION_OF_IMMUNE_EFFECTOR_PROCESS
GOBP_NEGATIVE_REGULATION_OF_IMMUNE_RESPONSE
GOBP_NEGATIVE_REGULATION_OF_IMMUNE_SYSTEM_PROCESS
GOBP_NEGATIVE_REGULATION_OF_LEUKOCYTE_APOPTOTIC_PROCESS
GOBP_NEGATIVE_REGULATION_OF_LEUKOCYTE_CELL_CELL_ADHESION
GOBP_NEGATIVE_REGULATION_OF_LEUKOCYTE_MEDIATED_IMMUNITY
GOBP_NEGATIVE_REGULATION_OF_LEUKOCYTE_PROLIFERATION
GOBP_NEGATIVE_REGULATION_OF_LYMPHOCYTE_ACTIVATION
GOBP_NEGATIVE_REGULATION_OF_LYMPHOCYTE_DIFFERENTIATION
GOBP_NEGATIVE_REGULATION_OF_LYMPHOCYTE_MEDIATED_IMMUNITY
GOBP_NEGATIVE_REGULATION_OF_MACROPHAGE_ACTIVATION
GOBP_NEGATIVE_REGULATION_OF_MONONUCLEAR_CELL_MIGRATION
GOBP_NEGATIVE_REGULATION_OF_MYELOID_LEUKOCYTE_DIFFERENTIATION
GOBP_NEGATIVE_REGULATION_OF_PRODUCTION_OF_MOLECULAR_MEDIATOR_OF_IMMUNE_RESPONSE
GOBP_NEGATIVE_REGULATION_OF_TOLL_LIKE_RECEPTOR_SIGNALING_PATHWAY
GOBP_NEGATIVE_REGULATION_OF_T_CELL_DIFFERENTIATION
GOBP_NEGATIVE_REGULATION_OF_T_CELL_MEDIATED_IMMUNITY
GOBP_NEGATIVE_REGULATION_OF_T_CELL_PROLIFERATION
GOBP_NEGATIVE_REGULATION_OF_T_CELL_RECEPTOR_SIGNALING_PATHWAY
GOBP_NEUTROPHIL_ACTIVATION_INVOLVED_IN_IMMUNE_RESPONSE
GOBP_NEUTROPHIL_CHEMOTAXIS
GOBP_NEUTROPHIL_HOMEOSTASIS
GOBP_NEUTROPHIL_MEDIATED_IMMUNITY
GOBP_NEUTROPHIL_MIGRATION
GOBP_POSITIVE_REGULATION_OF_ALPHA_BETA_T_CELL_PROLIFERATION
GOBP_POSITIVE_REGULATION_OF_ANTIGEN_RECEPTOR_MEDIATED_SIGNALING_PATHWAY
GOBP_POSITIVE_REGULATION_OF_B_CELL_ACTIVATION
GOBP_POSITIVE_REGULATION_OF_CD4_POSITIVE_ALPHA_BETA_T_CELL_ACTIVATION
GOBP_POSITIVE_REGULATION_OF_CD4_POSITIVE_ALPHA_BETA_T_CELL_DIFFERENTIATION
GOBP_POSITIVE_REGULATION_OF_CELLULAR_RESPONSE_TO_TRANSFORMING_GROWTH_FACTOR_BETA_STIMULUS
GOBP_POSITIVE_REGULATION_OF_CYTOKINE_PRODUCTION_INVOLVED_IN_IMMUNE_RESPONSE
GOBP_POSITIVE_REGULATION_OF_DEFENSE_RESPONSE
GOBP_POSITIVE_REGULATION_OF_FIBROBLAST_PROLIFERATION
GOBP_POSITIVE_REGULATION_OF_IMMUNE_EFFECTOR_PROCESS
GOBP_POSITIVE_REGULATION_OF_IMMUNE_RESPONSE

GOBP_POSITIVE_REGULATION_OF_LEUKOCYTE_ADHESION_TO_VASCULAR_ENDOTHELIAL_CELL
GOBP_POSITIVE_REGULATION_OF_LEUKOCYTE_APOPTOTIC_PROCESS
GOBP_POSITIVE_REGULATION_OF_LEUKOCYTE_CELL_CELL_ADHESION
GOBP_POSITIVE_REGULATION_OF_LEUKOCYTE_CHEMOTAXIS
GOBP_POSITIVE_REGULATION_OF_LEUKOCYTE_DEGRANULATION
GOBP_POSITIVE_REGULATION_OF_LEUKOCYTE_MEDIATED_IMMUNITY
GOBP_POSITIVE_REGULATION_OF_LEUKOCYTE_MIGRATION
GOBP_POSITIVE_REGULATION_OF_LEUKOCYTE_PROLIFERATION
GOBP_POSITIVE_REGULATION_OF_LYMPHOCYTE_DIFFERENTIATION
GOBP_POSITIVE_REGULATION_OF_LYMPHOCYTE_MEDIATED_IMMUNITY
GOBP_POSITIVE_REGULATION_OF_LYMPHOCYTE_MIGRATION
GOBP_POSITIVE_REGULATION_OF_MACROPHAGE_CHEMOTAXIS
GOBP_POSITIVE_REGULATION_OF_MACROPHAGE_MIGRATION
GOBP_POSITIVE_REGULATION_OF_MONOCYTE_CHEMOTAXIS
GOBP_POSITIVE_REGULATION_OF_MONONUCLEAR_CELL_MIGRATION
GOBP_POSITIVE_REGULATION_OF_MYELOID_CELL_DIFFERENTIATION
GOBP_POSITIVE_REGULATION_OF_MYELOID_LEUKOCYTE_DIFFERENTIATION
GOBP_POSITIVE_REGULATION_OF_MYELOID_LEUKOCYTE_MEDIATED_IMMUNITY
GOBP_POSITIVE_REGULATION_OF_NATURAL_KILLER_CELL_ACTIVATION
GOBP_POSITIVE_REGULATION_OF_NATURAL_KILLER_CELL_MEDIATED_CYTOTOXICITY
GOBP_POSITIVE_REGULATION_OF_REGULATORY_T_CELL_DIFFERENTIATION
GOBP_POSITIVE_REGULATION_OF_TOLL_LIKE_RECEPTOR_SIGNALING_PATHWAY
GOBP_POSITIVE_REGULATION_OF_T_CELL_CYTOKINE_PRODUCTION
GOBP_POSITIVE_REGULATION_OF_T_CELL_MEDIATED_CYTOTOXICITY
GOBP_POSITIVE_REGULATION_OF_T_CELL_MEDIATED_IMMUNITY
GOBP_POSITIVE_REGULATION_OF_T_CELL_MIGRATION
GOBP_POSITIVE_REGULATION_OF_T_CELL_PROLIFERATION
GOBP_POSITIVE_REGULATION_OF_T_HELPER_CELL_DIFFERENTIATION
GOBP_POSITIVE_REGULATION_OF_VIRAL_GENOME_REPLICATION
GOBP_POSITIVE_T_CELL_SELECTION
GOBP_PRODUCTION_OF_MOLECULAR_MEDIATOR_INVOLVED_IN_INFLAMMATORY_RESPONSE
GOBP_PRODUCTION_OF_MOLECULAR_MEDIATOR_OF_IMMUNE_RESPONSE
GOBP_PROGRAMMED_NECROTIC_CELL_DEATH
GOBP_REGULATION_OF_ADAPTIVE_IMMUNE_RESPONSE
GOBP_REGULATION_OF_ALPHA_BETA_T_CELL_ACTIVATION
GOBP_REGULATION_OF_ALPHA_BETA_T_CELL_DIFFERENTIATION
GOBP_REGULATION_OF_ANTIGEN_RECEPTOR_MEDIATED_SIGNALING_PATHWAY
GOBP_REGULATION_OF_B_CELL_ACTIVATION
GOBP_REGULATION_OF_B_CELL_DIFFERENTIATION
GOBP_REGULATION_OF_B_CELL_MEDIATED_IMMUNITY
GOBP_REGULATION_OF_B_CELL_PROLIFERATION
GOBP_REGULATION_OF_B_CELL_RECEPTOR_SIGNALING_PATHWAY
GOBP_REGULATION_OF_CD4_POSITIVE_ALPHA_BETA_T_CELL_ACTIVATION
GOBP_REGULATION_OF_CD4_POSITIVE_ALPHA_BETA_T_CELL_DIFFERENTIATION
GOBP_REGULATION_OF_CD8_POSITIVE_ALPHA_BETA_T_CELL_ACTIVATION
GOBP_REGULATION_OF_CELLULAR_RESPONSE_TO_TRANSFORMING_GROWTH_FACTOR_BETA_STIMULUS
GOBP_REGULATION_OF_COMPLEMENT_ACTIVATION
GOBP_REGULATION_OF_DEFENSE_RESPONSE_TO_BACTERIUM
GOBP_REGULATION_OF_GRANULOCYTE_CHEMOTAXIS
GOBP_REGULATION_OF_GRANULOCYTE_DIFFERENTIATION
GOBP_REGULATION_OF_HORMONE_BIOSYNTHETIC_PROCESS
GOBP_REGULATION_OF_HUMORAL_IMMUNE_RESPONSE
GOBP_REGULATION_OF_HUMORAL_IMMUNE_RESPONSE_MEDIATED_BY_CIRCULATING_IMMUNOGLOBULIN

GOBP_REGULATION_OF_IMMUNE_EFFECTOR_PROCESS
GOBP_REGULATION_OF_INNATE_IMMUNE_RESPONSE
GOBP_REGULATION_OF_LEUKOCYTE_ADHESION_TO_VASCULAR_ENDOTHELIAL_CELL
GOBP_REGULATION_OF_LEUKOCYTE_APOPTOTIC_PROCESS
GOBP_REGULATION_OF_LEUKOCYTE_CHEMOTAXIS
GOBP_REGULATION_OF_LEUKOCYTE_DEGRANULATION
GOBP_REGULATION_OF_LEUKOCYTE_DIFFERENTIATION
GOBP_REGULATION_OF_LEUKOCYTE_MEDIATED_CYTOTOXICITY
GOBP_REGULATION_OF_LEUKOCYTE_MEDIATED_IMMUNITY
GOBP_REGULATION_OF_LEUKOCYTE_MIGRATION
GOBP_REGULATION_OF_LEUKOCYTE_PROLIFERATION
GOBP_REGULATION_OF_LYMPHOCYTE_ACTIVATION
GOBP_REGULATION_OF_LYMPHOCYTE_APOPTOTIC_PROCESS
GOBP_REGULATION_OF_LYMPHOCYTE_DIFFERENTIATION
GOBP_REGULATION_OF_LYMPHOCYTE_MEDIATED_IMMUNITY
GOBP_REGULATION_OF_LYMPHOCYTE_MIGRATION
GOBP_REGULATION_OF_MACROPHAGE_ACTIVATION
GOBP_REGULATION_OF_MACROPHAGE_CHEMOTAXIS
GOBP_REGULATION_OF_MACROPHAGE_DIFFERENTIATION
GOBP_REGULATION_OF_MACROPHAGE_MIGRATION
GOBP_REGULATION_OF_MAST_CELL_ACTIVATION
GOBP_REGULATION_OF_MAST_CELL_ACTIVATION_INVOLVED_IN_IMMUNE_RESPONSE
GOBP_REGULATION_OF_MONOCYTE_CHEMOTAXIS
GOBP_REGULATION_OF_MONOCYTE_DIFFERENTIATION
GOBP_REGULATION_OF_MONONUCLEAR_CELL_MIGRATION
GOBP_REGULATION_OF_MYELOID_CELL_APOPTOTIC_PROCESS
GOBP_REGULATION_OF_MYELOID_CELL_DIFFERENTIATION
GOBP_REGULATION_OF_MYELOID_LEUKOCYTE_DIFFERENTIATION
GOBP_REGULATION_OF_MYELOID_LEUKOCYTE_MEDIATED_IMMUNITY
GOBP_REGULATION_OF_NATURAL_KILLER_CELL_ACTIVATION
GOBP_REGULATION_OF_NATURAL_KILLER_CELL_MEDIATED_IMMUNITY
GOBP_REGULATION_OF_NECROTIC_CELL_DEATH
GOBP_REGULATION_OF_NEUTROPHIL_MIGRATION
GOBP_REGULATION_OF_PRODUCTION_OF_MOLECULAR_MEDIATOR_OF_IMMUNE_RESPONSE
GOBP_REGULATION_OF_TOLERANCE_INDUCTION
GOBP_REGULATION_OF_TOLL_LIKE_RECEPTOR_SIGNALING_PATHWAY
GOBP_REGULATION_OF_T_CELL_ACTIVATION
GOBP_REGULATION_OF_T_CELL_APOPTOTIC_PROCESS
GOBP_REGULATION_OF_T_CELL_DIFFERENTIATION
GOBP_REGULATION_OF_T_CELL_DIFFERENTIATION_IN_THYMUS
GOBP_REGULATION_OF_T_CELL_MEDIATED_CYTOTOXICITY
GOBP_REGULATION_OF_T_CELL_MEDIATED_IMMUNITY
GOBP_REGULATION_OF_T_CELL_MIGRATION
GOBP_REGULATION_OF_T_CELL_PROLIFERATION
GOBP_REGULATION_OF_T_CELL_RECEPTOR_SIGNALING_PATHWAY
GOBP_REGULATION_OF_T_HELPER_17_TYPE_IMMUNE_RESPONSE
GOBP_REGULATION_OF_T_HELPER_CELL_DIFFERENTIATION
GOBP_REGULATORY_T_CELL_DIFFERENTIATION
GOBP_RESPONSE_TO_MOLECULE_OF_BACTERIAL_ORIGIN
GOBP_RESPONSE_TO_VIRUS
GOBP_TOLERANCE_INDUCTION
GOBP_TOLL_LIKE_RECEPTOR_2_SIGNALING_PATHWAY
GOBP_TOLL_LIKE_RECEPTOR_3_SIGNALING_PATHWAY

GOBP_TOLL_LIKE_RECEPTOR_4_SIGNALING_PATHWAY
GOBP_TOLL_LIKE_RECEPTOR_SIGNALING_PATHWAY
GOBP_TRANSCYTOSIS
GOBP_TYPE_2_IMMUNE_RESPONSE
GOBP_T_CELL_ACTIVATION
GOBP_T_CELL_ACTIVATION_INVOLVED_IN_IMMUNE_RESPONSE
GOBP_T_CELL_APOPTOTIC_PROCESS
GOBP_T_CELL_CYTOKINE_PRODUCTION
GOBP_T_CELL_DIFFERENTIATION
GOBP_T_CELL_DIFFERENTIATION_INVOLVED_IN_IMMUNE_RESPONSE
GOBP_T_CELL_DIFFERENTIATION_IN_THYMUS
GOBP_T_CELL_HOMEOSTASIS
GOBP_T_CELL_LINEAGE_COMMITMENT
GOBP_T_CELL_MEDIATED_CYTOTOXICITY
GOBP_T_CELL_MEDIATED_IMMUNITY
GOBP_T_CELL_MIGRATION
GOBP_T_CELL_PROLIFERATION
GOBP_T_CELL_RECEPTOR_SIGNALING_PATHWAY
GOBP_T_CELL_SELECTION
GOBP_T_HELPER_17_CELL_DIFFERENTIATION
GOBP_T_HELPER_17_TYPE_IMMUNE_RESPONSE
GOBP_T_HELPER_CELL_LINEAGE_COMMITMENT
GOBP_CELLULAR_DEFENSE_RESPONSE
KEGG_FC_EPSILON_RI_SIGNALING_PATHWAY
KEGG_FC_GAMMA_R_MEDIATED_PHAGOCYTOSIS
KEGG_LEUKOCYTE_TRANSENDOTHELIAL_MIGRATION
KEGG_NATURAL_KILLER_CELL_MEDIATED_CYTOTOXICITY
KEGG_NEUROTROPHIN_SIGNALING_PATHWAY
KEGG_NOD_LIKE_RECEPTOR_SIGNALING_PATHWAY
KEGG_TOLL_LIKE_RECEPTOR_SIGNALING_PATHWAY
KEGG_T_CELL_RECEPTOR_SIGNALING_PATHWAY

# MSigDB annotations - INFLAMMATION
GOBP_ACTIVATION_OF_CYSTEINE_TYPE_ENDOPEPTIDASE_ACTIVITY_INVOLVED_IN_APOPTOTIC_PROCESS
GOBP_ACTIVATION_OF_GTPASE_ACTIVITY
GOBP_ACTIVATION_OF_PHOSPHOLIPASE_C_ACTIVITY
GOBP_ACUTE_INFLAMMATORY_RESPONSE_TO_ANTIGENIC_STIMULUS
GOBP_CYTOKINE_MEDIATED_SIGNALING_PATHWAY
GOBP_INFLAMMATORY_CELL_APOPTOTIC_PROCESS
GOBP_INFLAMMATORY_RESPONSE_TO_ANTIGENIC_STIMULUS
GOBP_INTERFERON_GAMMA_PRODUCTION
GOBP_INTERLEUKIN_10_PRODUCTION
GOBP_INTERLEUKIN_12_PRODUCTION
GOBP_INTERLEUKIN_1_MEDIATED_SIGNALING_PATHWAY
GOBP_INTERLEUKIN_1_PRODUCTION
GOBP_INTERLEUKIN_2_PRODUCTION
GOBP_INTERLEUKIN_4_PRODUCTION
GOBP_INTERLEUKIN_5_PRODUCTION
GOBP_INTERLEUKIN_6_MEDIATED_SIGNALING_PATHWAY
GOBP_INTERLEUKIN_6_PRODUCTION
GOBP_INTERLEUKIN_8_PRODUCTION
GOBP_I_KAPPAB_KINASE_NF_KAPPAB_SIGNALING

GOBP_JNK_CASCADE
GOBP_NEGATIVE_REGULATION_OF_CYTOKINE_PRODUCTION
GOBP_NEGATIVE_REGULATION_OF_INFLAMMATORY_RESPONSE
GOBP_NEGATIVE_REGULATION_OF_INTERLEUKIN_10_PRODUCTION
GOBP_NEGATIVE_REGULATION_OF_INTERLEUKIN_1_BETA_PRODUCTION
GOBP_NEGATIVE_REGULATION_OF_INTERLEUKIN_1_PRODUCTION
GOBP_NEGATIVE_REGULATION_OF_INTERLEUKIN_2_PRODUCTION
GOBP_NEGATIVE_REGULATION_OF_INTERLEUKIN_6_PRODUCTION
GOBP_NEGATIVE_REGULATION_OF_INTERLEUKIN_8_PRODUCTION
GOBP_NEGATIVE_REGULATION_OF_TRANSFORMING_GROWTH_FACTOR_BETA_RECEPTOR_SIGNALING_PATHWAY
GOBP_NEGATIVE_REGULATION_OF_TUMOR_NECROSIS_FACTOR_SUPERFAMILY_CYTOKINE_PRODUCTION
GOBP_NIK_NF_KAPPAB_SIGNALING
GOBP_POSITIVE_REGULATION_OF_CELLULAR_RESPONSE_TO_TRANSFORMING_GROWTH_FACTOR_BETA_STIMULUS
GOBP_POSITIVE_REGULATION_OF_CYTOKINE_PRODUCTION
GOBP_POSITIVE_REGULATION_OF_INFLAMMATORY_RESPONSE
GOBP_POSITIVE_REGULATION_OF_INTERFERON_GAMMA_PRODUCTION
GOBP_POSITIVE_REGULATION_OF_INTERLEUKIN_10_PRODUCTION
GOBP_POSITIVE_REGULATION_OF_INTERLEUKIN_12_PRODUCTION
GOBP_POSITIVE_REGULATION_OF_INTERLEUKIN_1_BETA_PRODUCTION
GOBP_POSITIVE_REGULATION_OF_INTERLEUKIN_1_PRODUCTION
GOBP_POSITIVE_REGULATION_OF_INTERLEUKIN_2_PRODUCTION
GOBP_POSITIVE_REGULATION_OF_INTERLEUKIN_4_PRODUCTION
GOBP_POSITIVE_REGULATION_OF_INTERLEUKIN_6_PRODUCTION
GOBP_POSITIVE_REGULATION_OF_INTERLEUKIN_8_PRODUCTION
GOBP_POSITIVE_REGULATION_OF_RESPONSE_TO_CYTOKINE_STIMULUS
GOBP_POSITIVE_REGULATION_OF_TOLL_LIKE_RECEPTOR_SIGNALING_PATHWAY
GOBP_POSITIVE_REGULATION_OF_TUMOR_NECROSIS_FACTOR_SUPERFAMILY_CYTOKINE_PRODUCTION
GOBP_PRODUCTION_OF_MOLECULAR_MEDIATOR_INVOLVED_IN_INFLAMMATORY_RESPONSE
GOBP_PRODUCTION_OF_MOLECULAR_MEDIATOR_OF_IMMUNE_RESPONSE
GOBP_PROGRAMMED_NECROTIC_CELL_DEATH
GOBP_REGULATION_OF_CELLULAR_RESPONSE_TO_TRANSFORMING_GROWTH_FACTOR_BETA_STIMULUS
GOBP_REGULATION_OF_INFLAMMATORY_RESPONSE
GOBP_REGULATION_OF_INNATE_IMMUNE_RESPONSE
GOBP_REGULATION_OF_PROGRAMMED_NECROTIC_CELL_DEATH
GOBP_REGULATION_OF_RESPONSE_TO_CYTOKINE_STIMULUS
GOBP_CELLULAR_RESPONSE_TO_INTERFERON_GAMMA
GOBP_RESPONSE_TO_CHEMOKINE
GOBP_RESPONSE_TO_INTERFERON_GAMMA
GOBP_RESPONSE_TO_INTERLEUKIN_6
GOBP_RESPONSE_TO_ISCHEMIA
GOBP_RESPONSE_TO_LECTIN
GOBP_RESPONSE_TO_PROGESTERONE
GOBP_RESPONSE_TO_PROSTAGLANDIN_E
GOBP_RESPONSE_TO_TRANSFORMING_GROWTH_FACTOR_BETA
GOBP_RESPONSE_TO_TUMOR_NECROSIS_FACTOR
GOBP_TRANSFORMING_GROWTH_FACTOR_BETA_RECEPTOR_SIGNALING_PATHWAY
GOBP_TUMOR_NECROSIS_FACTOR_SUPERFAMILY_CYTOKINE_PRODUCTION
GOBP_T_CELL_APOPTOTIC_PROCESS
GOBP_T_CELL_CYTOKINE_PRODUCTION
GOBP_NEGATIVE_REGULATION_OF_FIBROBLAST_GROWTH_FACTOR_RECEPTOR_SIGNALING_PATHWAY
GOBP_POSITIVE_REGULATION_OF_I_KAPPAB_KINASE_NF_KAPPAB_SIGNALING
GOBP_ADRENERGIC_RECEPTOR_SIGNALING_PATHWAY
GOBP_REGULATION_OF_SMAD_PROTEIN_SIGNAL_TRANSDUCTION

GOBP_REGULATION_OF_SMALL_GTPASE_MEDIATED_SIGNAL_TRANSDUCTION
KEGG_JAK_STAT_SIGNALING_PATHWAY
KEGG_CYTOKINE_CYTOKINE_RECEPTOR_INTERACTION
HALLMARK_IL2_STAT5_SIGNALING
HALLMARK_IL6_JAK_STAT3_SIGNALING
HALLMARK_INFLAMMATORY_RESPONSE
HALLMARK_INTERFERON_GAMMA_RESPONSE
HALLMARK_TGF_BETA_SIGNALING

# MSigDB annotations - MAPK PATHWAY
GOBP_ERK1_AND_ERK2_CASCADE
GOBP_NEGATIVE_REGULATION_OF_ERK1_AND_ERK2_CASCADE
GOBP_NEGATIVE_REGULATION_OF_KINASE_ACTIVITY
GOBP_NEGATIVE_REGULATION_OF_MAPK_CASCADE
GOBP_NEGATIVE_REGULATION_OF_MAP_KINASE_ACTIVITY
GOBP_P38MAPK_CASCADE
GOBP_POSITIVE_REGULATION_OF_ERK1_AND_ERK2_CASCADE
GOBP_POSITIVE_REGULATION_OF_JNK_CASCADE
GOBP_POSITIVE_REGULATION_OF_JUN_KINASE_ACTIVITY
GOBP_POSITIVE_REGULATION_OF_KINASE_ACTIVITY
GOBP_POSITIVE_REGULATION_OF_MAPK_CASCADE
GOBP_POSITIVE_REGULATION_OF_MAP_KINASE_ACTIVITY
GOBP_POSITIVE_REGULATION_OF_P38MAPK_CASCADE
GOBP_RAC_PROTEIN_SIGNAL_TRANSDUCTION
GOBP_RAS_PROTEIN_SIGNAL_TRANSDUCTION
GOBP_REGULATION_OF_JNK_CASCADE
GOBP_REGULATION_OF_JUN_KINASE_ACTIVITY
GOBP_REGULATION_OF_MAP_KINASE_ACTIVITY
GOBP_REGULATION_OF_P38MAPK_CASCADE
GOBP_REGULATION_OF_PROTEIN_TYROSINE_KINASE_ACTIVITY
GOBP_REGULATION_OF_RAS_PROTEIN_SIGNAL_TRANSDUCTION
GOBP_RECEPTOR_SIGNALING_PATHWAY_VIA_STAT
KEGG_MAPK_SIGNALING_PATHWAY
HALLMARK_KRAS_SIGNALING_UP

# MSigDB annotations - MEMBRANE TRANSPORT
GOBP_ENDOCYTIC_RECYCLING
GOBP_CLATHRIN_DEPENDENT_ENDOCYTOSIS
GOBP_EXOCYTOSIS
GOBP_NEGATIVE_REGULATION_OF_EXOCYTOSIS
GOBP_NEGATIVE_REGULATION_OF_TRANSMEMBRANE_TRANSPORT
GOBP_ORGANELLE_FISSION
GOBP_PHAGOCYTOSIS
GOBP_PHAGOLYSOSOME_ASSEMBLY
GOBP_PHAGOSOME_MATURATION
GOBP_PINOCYTOSIS
GOBP_POSITIVE_REGULATION_OF_AUTOPHAGY
GOBP_POSITIVE_REGULATION_OF_ENDOCYTOSIS
GOBP_POSITIVE_REGULATION_OF_ORGANELLE_ASSEMBLY
GOBP_POSITIVE_REGULATION_OF_ORGANELLE_ORGANIZATION
GOBP_POSITIVE_REGULATION_OF_PHAGOCYTOSIS

GOBP_POSITIVE_REGULATION_OF_TRANSMEMBRANE_TRANSPORT
GOBP_RECEPTOR_INTERNALIZATION
GOBP_RECEPTOR_MEDIATED_ENDOCYTOSIS
GOBP_REGULATION_OF_ENDOCYTIC_RECYCLING
GOBP_REGULATION_OF_ENDOCYTOSIS
GOBP_REGULATION_OF_PHAGOCYTOSIS
GOBP_CHAPERONE_MEDIATED_AUTOPHAGY
GOBP_EXOCRINE_SYSTEM_DEVELOPMENT
GOBP_EXOCRINE_SYSTEM_DEVELOPMENT
GOBP_NEGATIVE_REGULATION_OF_TRANSPORT
GOBP_PLASMA_MEMBRANE_ORGANIZATION
GOBP_PLASMA_MEMBRANE_REPAIR
GOBP_PLASMA_MEMBRANE_TUBULATION
GOBP_POSITIVE_REGULATION_OF_INTRACELLULAR_TRANSPORT
GOBP_POSITIVE_REGULATION_OF_PROTEIN_SECRETION
GOBP_POSITIVE_REGULATION_OF_REGULATED_SECRETORY_PATHWAY
GOBP_POSITIVE_REGULATION_OF_SECRETION
GOBP_POSITIVE_REGULATION_OF_VACUOLE_ORGANIZATION
GOBP_REGULATED_EXOCYTOSIS
GOBP_REGULATION_OF_MEMBRANE_INVAGINATION
GOBP_VACUOLAR_LOCALIZATION
GOBP_VACUOLE_ORGANIZATION
GOBP_MEMBRANE_DEPOLARIZATION
GOBP_MEMBRANE_INVAGINATION
GOBP_NON_MEMBRANE_BOUNDED_ORGANELLE_ASSEMBLY
GOBP_REGULATION_OF_MEMBRANE_LIPID_DISTRIBUTION
GOBP_REGULATION_OF_RECEPTOR_MEDIATED_ENDOCYTOSIS
GOBP_REGULATION_OF_RUFFLE_ASSEMBLY
KEGG_LYSOSOME
KEGG_ENDOCYTOSIS

# MSigDB annotations - METABOLISM
GOBP_AMINE_METABOLIC_PROCESS
GOBP_AMINOGLYCAN_BIOSYNTHETIC_PROCESS
GOBP_AMINOGLYCAN_CATABOLIC_PROCESS
GOBP_AMINOGLYCAN_METABOLIC_PROCESS
GOBP_AMYLOID_BETA_CLEARANCE
GOBP_APOPTOTIC_CELL_CLEARANCE
GOBP_CELLULAR_LIPID_CATABOLIC_PROCESS
GOBP_CHOLESTEROL_STORAGE
GOBP_DEOXYRIBOSE_PHOSPHATE_METABOLIC_PROCESS
GOBP_FATTY_ACID_TRANSMEMBRANE_TRANSPORT
GOBP_GLYCEROLIPID_CATABOLIC_PROCESS
GOBP_GLYCEROLIPID_METABOLIC_PROCESS
GOBP_GLYCEROPHOSPHOLIPID_CATABOLIC_PROCESS
GOBP_GLYCOSAMINOGLYCAN_CATABOLIC_PROCESS
GOBP_HEPARAN_SULFATE_PROTEOGLYCAN_METABOLIC_PROCESS
GOBP_HEPARIN_METABOLIC_PROCESS
GOBP_INNER_MITOCHONDRIAL_MEMBRANE_ORGANIZATION
GOBP_INOSITOL_LIPID_MEDIATED_SIGNALING
GOBP_INOSITOL_PHOSPHATE_CATABOLIC_PROCESS
GOBP_INOSITOL_PHOSPHATE_MEDIATED_SIGNALING

GOBP_INTRACELLULAR_LIPID_TRANSPORT
GOBP_LIPID_CATABOLIC_PROCESS
GOBP_LIPID_LOCALIZATION
GOBP_LIPID_STORAGE
GOBP_LIPID_TRANSLOCATION
GOBP_LONG_CHAIN_FATTY_ACID_TRANSPORT
GOBP_MITOCHONDRIAL_GENE_EXPRESSION
GOBP_MITOCHONDRIAL_MEMBRANE_ORGANIZATION
GOBP_MITOCHONDRIAL_RNA_METABOLIC_PROCESS
GOBP_MITOCHONDRIAL_TRANSLATION
GOBP_MONOVALENT_INORGANIC_CATION_HOMEOSTASIS
GOBP_MUCOPOLYSACCHARIDE_METABOLIC_PROCESS
GOBP_NEGATIVE_REGULATION_OF_ADENYLATE_CYCLASE_ACTIVITY
GOBP_NEGATIVE_REGULATION_OF_GTPASE_ACTIVITY
GOBP_NEGATIVE_REGULATION_OF_PHOSPHORUS_METABOLIC_PROCESS
GOBP_NEGATIVE_REGULATION_OF_REACTIVE_OXYGEN_SPECIES_BIOSYNTHETIC_PROCESS
GOBP_NEGATIVE_REGULATION_OF_REGULATED_SECRETORY_PATHWAY
GOBP_NEUTRAL_LIPID_BIOSYNTHETIC_PROCESS
GOBP_NEUTRAL_LIPID_METABOLIC_PROCESS
GOBP_PHOSPHOLIPID_CATABOLIC_PROCESS
GOBP_PHOSPHOLIPID_DEPHOSPHORYLATION
GOBP_PHOSPHOLIPID_TRANSPORT
GOBP_POSITIVE_REGULATION_OF_LIPASE_ACTIVITY
GOBP_POSITIVE_REGULATION_OF_LIPID_LOCALIZATION
GOBP_POSITIVE_REGULATION_OF_LIPID_STORAGE
GOBP_POSITIVE_REGULATION_OF_LIPID_TRANSPORT
GOBP_POSITIVE_REGULATION_OF_REACTIVE_OXYGEN_SPECIES_BIOSYNTHETIC_PROCESS
GOBP_POSITIVE_REGULATION_OF_STEROL_TRANSPORT
GOBP_PROTEOGLYCAN_BIOSYNTHETIC_PROCESS
GOBP_PROTEOGLYCAN_METABOLIC_PROCESS
GOBP_PSEUDOURIDINE_SYNTHESIS
GOBP_REACTIVE_OXYGEN_SPECIES_BIOSYNTHETIC_PROCESS
GOBP_REGULATION_OF_GLYCOPROTEIN_METABOLIC_PROCESS
GOBP_REGULATION_OF_GTPASE_ACTIVITY
GOBP_REGULATION_OF_LIPASE_ACTIVITY
GOBP_REGULATION_OF_LIPID_KINASE_ACTIVITY
GOBP_REGULATION_OF_LIPID_STORAGE
GOBP_RESPONSE_TO_AMINO_ACID_STARVATION
GOBP_TRIGLYCERIDE_METABOLIC_PROCESS
GOBP_VITAMIN_CATABOLIC_PROCESS
GOBP_MEMBRANE_LIPID_CATABOLIC_PROCESS
GOBP_RECEPTOR_METABOLIC_PROCESS
HALLMARK_OXIDATIVE_PHOSPHORYLATION
KEGG_ALPHA_LINOLENIC_ACID_METABOLISM
KEGG_ETHER_LIPID_METABOLISM
KEGG_GLYCOSAMINOGLYCAN_BIOSYNTHESIS_CHONDROITIN_SULFATE
KEGG_GLYCOSAMINOGLYCAN_DEGRADATION
KEGG_GLYCOSPHINGOLIPID_BIOSYNTHESIS_GANGLIO_SERIES

# MSigDB annotations - MIGRATION
GOBP_CELL_ADHESION_MEDIATED_BY_INTEGRIN
GOBP_CELL_CELL_ADHESION_MEDIATED_BY_INTEGRIN

GOBP_CELL_CELL_JUNCTION_ASSEMBLY
GOBP_CELL_CELL_JUNCTION_ORGANIZATION
GOBP_FOCAL_ADHESION_ASSEMBLY
GOBP_CELL_CHEMOTAXIS
GOBP_CELL_MATRIX_ADHESION
GOBP_CELL_MIGRATION_INVOLVED_IN_SPROUTING_ANGIOGENESIS
GOBP_CELL_PROJECTION_ASSEMBLY
GOBP_CELL_RECOGNITION
GOBP_CELL_SUBSTRATE_ADHESION
GOBP_CELL_SUBSTRATE_JUNCTION_ORGANIZATION
GOBP_CLATHRIN_COAT_ASSEMBLY
GOBP_COLLAGEN_ACTIVATED_SIGNALING_PATHWAY
GOBP_COLLAGEN_CATABOLIC_PROCESS
GOBP_COLLAGEN_METABOLIC_PROCESS
GOBP_EXTRACELLULAR_MATRIX_ASSEMBLY
GOBP_INTEGRIN_ACTIVATION
GOBP_INTEGRIN_MEDIATED_SIGNALING_PATHWAY
GOBP_INTERMEDIATE_FILAMENT_BASED_PROCESS
GOBP_INTERMEDIATE_FILAMENT_ORGANIZATION
GOBP_LAMELLIPODIUM_ASSEMBLY
GOBP_LAMELLIPODIUM_MORPHOGENESIS
GOBP_LAMELLIPODIUM_ORGANIZATION
GOBP_MAINTENANCE_OF_CELL_POLARITY
GOBP_MAINTENANCE_OF_LOCATION
GOBP_MAINTENANCE_OF_LOCATION_IN_CELL
GOBP_NEGATIVE_CHEMOTAXIS
GOBP_NEGATIVE_REGULATION_OF_CELL_ADHESION
GOBP_NEGATIVE_REGULATION_OF_CELL_CELL_ADHESION
GOBP_NEGATIVE_REGULATION_OF_CELL_JUNCTION_ASSEMBLY
GOBP_NEGATIVE_REGULATION_OF_CELL_MATRIX_ADHESION
GOBP_NEGATIVE_REGULATION_OF_CELL_MIGRATION_INVOLVED_IN_SPROUTING_ANGIOGENESIS
GOBP_NEGATIVE_REGULATION_OF_CELL_PROJECTION_ORGANIZATION
GOBP_NEGATIVE_REGULATION_OF_CELL_SUBSTRATE_ADHESION
GOBP_NEGATIVE_REGULATION_OF_CELL_SUBSTRATE_JUNCTION_ORGANIZATION
GOBP_NEGATIVE_REGULATION_OF_LOCOMOTION
GOBP_NEGATIVE_REGULATION_OF_PLASMA_MEMBRANE_BOUNDED_CELL_PROJECTION_ASSEMBLY
GOBP_POSITIVE_CHEMOTAXIS
GOBP_POSITIVE_REGULATION_OF_CELL_ADHESION
GOBP_POSITIVE_REGULATION_OF_CELL_CELL_ADHESION
GOBP_POSITIVE_REGULATION_OF_CELL_MATRIX_ADHESION
GOBP_POSITIVE_REGULATION_OF_CELL_PROJECTION_ORGANIZATION
GOBP_POSITIVE_REGULATION_OF_CELL_SUBSTRATE_ADHESION
GOBP_POSITIVE_REGULATION_OF_CELL_SUBSTRATE_JUNCTION_ORGANIZATION
GOBP_POSITIVE_REGULATION_OF_CHEMOTAXIS
GOBP_CELL_JUNCTION_ASSEMBLY
GOBP_CELL_JUNCTION_DISASSEMBLY
GOBP_SUBSTRATE_ADHESION_DEPENDENT_CELL_SPREADING
GOBP_REGULATION_OF_CHEMOTAXIS
GOBP_REGULATION_OF_EXTRACELLULAR_MATRIX_ORGANIZATION
GOBP_POSITIVE_REGULATION_OF_EXTRACELLULAR_MATRIX_ORGANIZATION
GOBP_REGULATION_OF_CELL_MATRIX_ADHESION
GOBP_REGULATION_OF_CELL_CELL_ADHESION
GOBP_REGULATION_OF_CELL_SUBSTRATE_ADHESION

GOBP_REGULATION_OF_CELL_SUBSTRATE_JUNCTION_ORGANIZATION
GOBP_SEMAPHORIN_PLEXIN_SIGNALING_PATHWAY
GOBP_SUBSTRATE_DEPENDENT_CELL_MIGRATION
KEGG_ADHERENS_JUNCTION
KEGG_ECM_RECEPTOR_INTERACTION
KEGG_FOCAL_ADHESION
KEGG_CELL_ADHESION_MOLECULES_CAMS

# MSigDB annotations - MOLECULAR PROCESSES
GOBP_CELLULAR_RESPONSE_TO_CAMP
GOBP_CELLULAR_RESPONSE_TO_COPPER_ION
GOBP_CELLULAR_RESPONSE_TO_EXTERNAL_STIMULUS
GOBP_CELLULAR_RESPONSE_TO_FLUID_SHEAR_STRESS
GOBP_CELLULAR_RESPONSE_TO_GAMMA_RADIATION
GOBP_CELLULAR_RESPONSE_TO_LIGHT_STIMULUS
GOBP_CELLULAR_RESPONSE_TO_LIPOPROTEIN_PARTICLE_STIMULUS
GOBP_CELLULAR_RESPONSE_TO_LOW_DENSITY_LIPOPROTEIN_PARTICLE_STIMULUS
GOBP_CELLULAR_RESPONSE_TO_MECHANICAL_STIMULUS
GOBP_CELLULAR_RESPONSE_TO_MOLECULE_OF_BACTERIAL_ORIGIN
GOBP_CELLULAR_RESPONSE_TO_OSMOTIC_STRESS
GOBP_CELLULAR_RESPONSE_TO_PEPTIDE
GOBP_CELLULAR_RESPONSE_TO_PROSTAGLANDIN_E_STIMULUS
GOBP_CELL_GROWTH
GOBP_CELL_KILLING
GOBP_CELL_VOLUME_HOMEOSTASIS
GOBP_CYTOPLASMIC_MICROTUBULE_ORGANIZATION
GOBP_CYTOPLASMIC_TRANSLATIONAL_INITIATION
GOBP_EPHRIN_RECEPTOR_SIGNALING_PATHWAY
GOBP_EXTRINSIC_APOPTOTIC_SIGNALING_PATHWAY_VIA_DEATH_DOMAIN_RECEPTORS
GOBP_FIBROBLAST_APOPTOTIC_PROCESS
GOBP_HETEROPHILIC_CELL_CELL_ADHESION_VIA_PLASMA_MEMBRANE_CELL_ADHESION_MOLECULES
GOBP_HOMEOSTASIS_OF_NUMBER_OF_CELLS
GOBP_HOMEOSTASIS_OF_NUMBER_OF_CELLS_WITHIN_A_TISSUE
GOBP_MAINTENANCE_OF_PROTEIN_LOCATION
GOBP_MOLTING_CYCLE
GOBP_NEGATIVE_REGULATION_OF_ANDROGEN_RECEPTOR_SIGNALING_PATHWAY
GOBP_NEGATIVE_REGULATION_OF_ANTIGEN_RECEPTOR_MEDIATED_SIGNALING_PATHWAY
GOBP_NEGATIVE_REGULATION_OF_CELL_ACTIVATION
GOBP_NEGATIVE_REGULATION_OF_CELL_KILLING
GOBP_NEGATIVE_REGULATION_OF_INTRACELLULAR_SIGNAL_TRANSDUCTION
GOBP_NEGATIVE_REGULATION_OF_PEPTIDYL_THREONINE_PHOSPHORYLATION
GOBP_NEGATIVE_REGULATION_OF_PEPTIDYL_TYROSINE_PHOSPHORYLATION
GOBP_NEGATIVE_REGULATION_OF_PHOSPHORYLATION
GOBP_NEGATIVE_REGULATION_OF_PROTEIN_MATURATION
GOBP_NEGATIVE_REGULATION_OF_PROTEIN_SERINE_THREONINE_KINASE_ACTIVITY
GOBP_NEGATIVE_REGULATION_OF_PROTEIN_TYROSINE_KINASE_ACTIVITY
GOBP_NEGATIVE_REGULATION_OF_RESPONSE_TO_EXTERNAL_STIMULUS
GOBP_NEGATIVE_REGULATION_OF_SMALL_GTPASE_MEDIATED_SIGNAL_TRANSDUCTION
GOBP_NEGATIVE_REGULATION_OF_SODIUM_ION_TRANSPORT
GOBP_NEGATIVE_REGULATION_OF_STEM_CELL_DIFFERENTIATION
GOBP_NEGATIVE_REGULATION_OF_TRANSFERASE_ACTIVITY
GOBP_OLIGOPEPTIDE_TRANSPORT

GOBP_ORGANOPHOSPHATE_CATABOLIC_PROCESS
GOBP_ORGANOPHOSPHATE_ESTER_TRANSPORT
GOBP_PATTERN_RECOGNITION_RECEPTOR_SIGNALING_PATHWAY
GOBP_PEPTIDE_CROSS_LINKING
GOBP_PEPTIDYL_THREONINE_MODIFICATION
GOBP_PEPTIDYL_TYROSINE_DEPHOSPHORYLATION
GOBP_PEPTIDYL_TYROSINE_MODIFICATION
GOBP_PHOSPHATIDYLCHOLINE_CATABOLIC_PROCESS
GOBP_PHOSPHATIDYLINOSITOL_3_KINASE_SIGNALING
GOBP_PHOSPHATIDYLINOSITOL_DEPHOSPHORYLATION
GOBP_PHOSPHATIDYLINOSITOL_PHOSPHATE_BIOSYNTHETIC_PROCESS
GOBP_PHOSPHOLIPASE_C_ACTIVATING_G_PROTEIN_COUPLED_RECEPTOR_SIGNALING_PATHWAY
GOBP_POSITIVE_REGULATION_OF_CELLULAR_COMPONENT_BIOGENESIS
GOBP_POSITIVE_REGULATION_OF_CELL_ACTIVATION
GOBP_POSITIVE_REGULATION_OF_CELL_KILLING
GOBP_POSITIVE_REGULATION_OF_CYSTEINE_TYPE_ENDOPEPTIDASE_ACTIVITY
GOBP_POSITIVE_REGULATION_OF_CYTOSOLIC_CALCIUM_ION_CONCENTRATION_INVOLVED_IN_PHOSPHOLIPASE_C_A
GOBP_POSITIVE_REGULATION_OF_DEPHOSPHORYLATION
GOBP_POSITIVE_REGULATION_OF_GTPASE_ACTIVITY
GOBP_POSITIVE_REGULATION_OF_ION_TRANSPORT
GOBP_POSITIVE_REGULATION_OF_NITRIC_OXIDE_SYNTHASE_BIOSYNTHETIC_PROCESS
GOBP_POSITIVE_REGULATION_OF_NUCLEOCYTOPLASMIC_TRANSPORT
GOBP_POSITIVE_REGULATION_OF_PATTERN_RECOGNITION_RECEPTOR_SIGNALING_PATHWAY
GOBP_POSITIVE_REGULATION_OF_PEPTIDASE_ACTIVITY
GOBP_POSITIVE_REGULATION_OF_PEPTIDYL_SERINE_PHOSPHORYLATION
GOBP_POSITIVE_REGULATION_OF_PEPTIDYL_TYROSINE_PHOSPHORYLATION
GOBP_POSITIVE_REGULATION_OF_PHOSPHATASE_ACTIVITY
GOBP_POSITIVE_REGULATION_OF_PHOSPHATIDYLINOSITOL_3_KINASE_SIGNALING
GOBP_POSITIVE_REGULATION_OF_PHOSPHOLIPASE_ACTIVITY
GOBP_POSITIVE_REGULATION_OF_PHOSPHOPROTEIN_PHOSPHATASE_ACTIVITY
GOBP_POSITIVE_REGULATION_OF_PLASMA_MEMBRANE_BOUNDED_CELL_PROJECTION_ASSEMBLY
GOBP_POSITIVE_REGULATION_OF_PROTEIN_CONTAINING_COMPLEX_ASSEMBLY
GOBP_POSITIVE_REGULATION_OF_PROTEIN_DEPHOSPHORYLATION
GOBP_POSITIVE_REGULATION_OF_PROTEIN_EXPORT_FROM_NUCLEUS
GOBP_POSITIVE_REGULATION_OF_PROTEIN_IMPORT
GOBP_POSITIVE_REGULATION_OF_PROTEIN_KINASE_ACTIVITY
GOBP_POSITIVE_REGULATION_OF_PROTEIN_LOCALIZATION_TO_CELL_PERIPHERY
GOBP_POSITIVE_REGULATION_OF_PROTEIN_LOCALIZATION_TO_CELL_SURFACE
GOBP_POSITIVE_REGULATION_OF_PROTEIN_LOCALIZATION_TO_MEMBRANE
GOBP_POSITIVE_REGULATION_OF_PROTEIN_LOCALIZATION_TO_NUCLEUS
GOBP_POSITIVE_REGULATION_OF_PROTEIN_POLYMERIZATION
GOBP_POSITIVE_REGULATION_OF_PROTEIN_SERINE_THREONINE_KINASE_ACTIVITY
GOBP_POSITIVE_REGULATION_OF_PROTEIN_TARGETING_TO_MEMBRANE
GOBP_POSITIVE_REGULATION_OF_PROTEIN_TYROSINE_KINASE_ACTIVITY
GOBP_POSITIVE_REGULATION_OF_PROTEOLYSIS
GOBP_POSITIVE_REGULATION_OF_RESPONSE_TO_EXTERNAL_STIMULUS
GOBP_POSITIVE_REGULATION_OF_RHO_PROTEIN_SIGNAL_TRANSDUCTION
GOBP_POSITIVE_REGULATION_OF_SMALL_GTPASE_MEDIATED_SIGNAL_TRANSDUCTION
GOBP_POSITIVE_REGULATION_OF_STRESS_ACTIVATED_PROTEIN_KINASE_SIGNALING_CASCADE
GOBP_POSITIVE_REGULATION_OF_TRANSMEMBRANE_RECEPTOR_PROTEIN_SERINE_THREONINE_KINASE_SIGNALING
GOBP_PROTEIN_ADP_RIBOSYLATION
GOBP_PROTEIN_AUTOPHOSPHORYLATION
GOBP_PROTEIN_DEGLYCOSYLATION

GOBP_PROTEIN_EXIT_FROM_ENDOPLASMIC_RETICULUM
GOBP_PROTEIN_K63_LINKED_UBIQUITINATION
GOBP_PROTEIN_KINASE_B_SIGNALING
GOBP_PROTEIN_LOCALIZATION_TO_CELL_SURFACE
GOBP_PROTEIN_LOCALIZATION_TO_CHROMATIN
GOBP_PROTEIN_LOCALIZATION_TO_CHROMOSOME
GOBP_PROTEIN_LOCALIZATION_TO_CHROMOSOME_CENTROMERIC_REGION
GOBP_PROTEIN_LOCALIZATION_TO_EXTRACELLULAR_REGION
GOBP_PROTEIN_LOCALIZATION_TO_NUCLEUS
GOBP_PROTEIN_LOCALIZATION_TO_PLASMA_MEMBRANE
GOBP_REGULATION_OF_CATION_TRANSMEMBRANE_TRANSPORT
GOBP_REGULATION_OF_CELLULAR_COMPONENT_SIZE
GOBP_REGULATION_OF_CELLULAR_EXTRAVASATION
GOBP_REGULATION_OF_CELLULAR_RESPONSE_TO_GROWTH_FACTOR_STIMULUS
GOBP_REGULATION_OF_CELL_JUNCTION_ASSEMBLY
GOBP_REGULATION_OF_CELL_KILLING
GOBP_REGULATION_OF_CELL_SHAPE
GOBP_REGULATION_OF_CELL_SIZE
GOBP_REGULATION_OF_CILIUM_ASSEMBLY
GOBP_REGULATION_OF_COLLAGEN_METABOLIC_PROCESS
GOBP_REGULATION_OF_CYSTEINE_TYPE_ENDOPEPTIDASE_ACTIVITY
GOBP_REGULATION_OF_CYTOPLASMIC_TRANSLATION
GOBP_REGULATION_OF_CYTOPLASMIC_TRANSPORT
GOBP_REGULATION_OF_DEPHOSPHORYLATION
GOBP_REGULATION_OF_EXTRINSIC_APOPTOTIC_SIGNALING_PATHWAY_VIA_DEATH_DOMAIN_RECEPTORS
GOBP_REGULATION_OF_HOMOTYPIC_CELL_CELL_ADHESION
GOBP_REGULATION_OF_INSULIN_RECEPTOR_SIGNALING_PATHWAY
GOBP_REGULATION_OF_METAL_ION_TRANSPORT
GOBP_REGULATION_OF_PATTERN_RECOGNITION_RECEPTOR_SIGNALING_PATHWAY
GOBP_REGULATION_OF_PEPTIDYL_SERINE_PHOSPHORYLATION
GOBP_REGULATION_OF_PEPTIDYL_TYROSINE_PHOSPHORYLATION
GOBP_REGULATION_OF_PHOSPHATASE_ACTIVITY
GOBP_REGULATION_OF_PHOSPHATIDYLINOSITOL_3_KINASE_ACTIVITY
GOBP_REGULATION_OF_PHOSPHATIDYLINOSITOL_3_KINASE_SIGNALING
GOBP_REGULATION_OF_PHOSPHOLIPASE_ACTIVITY
GOBP_REGULATION_OF_PHOSPHOLIPASE_C_ACTIVITY
GOBP_REGULATION_OF_PHOSPHOLIPID_TRANSPORT
GOBP_REGULATION_OF_PHOSPHOPROTEIN_PHOSPHATASE_ACTIVITY
GOBP_REGULATION_OF_PROTEIN_AUTOPHOSPHORYLATION
GOBP_REGULATION_OF_PROTEIN_CONTAINING_COMPLEX_ASSEMBLY
GOBP_REGULATION_OF_PROTEIN_CONTAINING_COMPLEX_DISASSEMBLY
GOBP_REGULATION_OF_PROTEIN_DEPHOSPHORYLATION
GOBP_REGULATION_OF_PROTEIN_DEPOLYMERIZATION
GOBP_REGULATION_OF_PROTEIN_EXIT_FROM_ENDOPLASMIC_RETICULUM
GOBP_REGULATION_OF_PROTEIN_IMPORT
GOBP_REGULATION_OF_PROTEIN_LOCALIZATION_TO_CELL_PERIPHERY
GOBP_REGULATION_OF_PROTEIN_LOCALIZATION_TO_CELL_SURFACE
GOBP_REGULATION_OF_PROTEIN_LOCALIZATION_TO_MEMBRANE
GOBP_REGULATION_OF_PROTEIN_LOCALIZATION_TO_NUCLEUS
GOBP_REGULATION_OF_PROTEIN_LOCALIZATION_TO_PLASMA_MEMBRANE
GOBP_REGULATION_OF_PROTEIN_MATURATION
GOBP_REGULATION_OF_PROTEIN_POLYMERIZATION
GOBP_REGULATION_OF_PROTEIN_SECRETION

GOBP_REGULATION_OF_PROTEIN_SERINE_THREONINE_KINASE_ACTIVITY
GOBP_REGULATION_OF_PROTEIN_TARGETING_TO_MEMBRANE
GOBP_REGULATION_OF_REACTIVE_OXYGEN_SPECIES_BIOSYNTHETIC_PROCESS
GOBP_REGULATION_OF_REGULATED_SECRETORY_PATHWAY
GOBP_REGULATION_OF_RESPONSE_TO_EXTRACELLULAR_STIMULUS
GOBP_REGULATION_OF_RESPONSE_TO_TUMOR_CELL
GOBP_REGULATION_OF_RHO_PROTEIN_SIGNAL_TRANSDUCTION
GOBP_REGULATION_OF_SEQUESTERING_OF_TRIGLYCERIDE
GOBP_REGULATION_OF_SMOOTHENED_SIGNALING_PATHWAY
GOBP_REGULATION_OF_SODIUM_ION_TRANSMEMBRANE_TRANSPORT
GOBP_REGULATION_OF_SODIUM_ION_TRANSPORT
GOBP_REGULATION_OF_STEM_CELL_DIFFERENTIATION
GOBP_REGULATION_OF_STEM_CELL_POPULATION_MAINTENANCE
GOBP_REGULATION_OF_STEROL_TRANSPORT
GOBP_REGULATION_OF_STRESS_ACTIVATED_PROTEIN_KINASE_SIGNALING_CASCADE
GOBP_REGULATION_OF_SUPEROXIDE_ANION_GENERATION
GOBP_REGULATION_OF_SUPRAMOLECULAR_FIBER_ORGANIZATION
GOBP_REGULATION_OF_TRANSMEMBRANE_RECEPTOR_PROTEIN_SERINE_THREONINE_KINASE_SIGNALING_PATHWA
GOBP_RESPONSE_TO_MANGANESE_ION
GOBP_RESPONSE_TO_MECHANICAL_STIMULUS
GOBP_RESPONSE_TO_OSMOTIC_STRESS
GOBP_RESPONSE_TO_OXYGEN_LEVELS
GOBP_SECOND_MESSENGER_MEDIATED_SIGNALING
GOBP_SMALL_GTPASE_MEDIATED_SIGNAL_TRANSDUCTION
GOBP_STEM_CELL_DIFFERENTIATION
GOBP_STRESS_ACTIVATED_PROTEIN_KINASE_SIGNALING_CASCADE
GOBP_SUPEROXIDE_ANION_GENERATION
GOBP_TISSUE_REMODELING
GOBP_TRANSMEMBRANE_RECEPTOR_PROTEIN_SERINE_THREONINE_KINASE_SIGNALING_PATHWAY
GOBP_ARF_PROTEIN_SIGNAL_TRANSDUCTION
GOBP_CELLULAR_COMPONENT_MAINTENANCE
GOBP_RHO_PROTEIN_SIGNAL_TRANSDUCTION
KEGG_PROTEASOME
GOBP_CALCINEURIN_MEDIATED_SIGNALING
GOBP_CALCIUM_ION_IMPORT
GOBP_CALCIUM_ION_TRANSMEMBRANE_TRANSPORT
GOBP_CALCIUM_ION_TRANSPORT
GOBP_CALCIUM_ION_TRANSPORT_INTO_CYTOSOL
GOBP_CALCIUM_MEDIATED_SIGNALING
GOBP_POSITIVE_REGULATION_OF_CALCIUM_ION_IMPORT
GOBP_POSITIVE_REGULATION_OF_CALCIUM_ION_TRANSPORT
GOBP_REGULATION_OF_CALCIUM_ION_IMPORT
GOBP_REGULATION_OF_CALCIUM_ION_TRANSMEMBRANE_TRANSPORT
GOBP_REGULATION_OF_CALCIUM_ION_TRANSMEMBRANE_TRANSPORTER_ACTIVITY
GOBP_REGULATION_OF_CALCIUM_ION_TRANSPORT
GOBP_REGULATION_OF_CALCIUM_ION_TRANSPORT_INTO_CYTOSOL
GOBP_REGULATION_OF_CALCIUM_MEDIATED_SIGNALING
GOBP_REGULATION_OF_CAMP_DEPENDENT_PROTEIN_KINASE_ACTIVITY
GOBP_REGULATION_OF_CAMP_MEDIATED_SIGNALING
GOBP_REGULATION_OF_RELEASE_OF_SEQUESTERED_CALCIUM_ION_INTO_CYTOSOL
GOBP_REGULATION_OF_VOLTAGE_GATED_CALCIUM_CHANNEL_ACTIVITY
GOBP_NEGATIVE_REGULATION_OF_CALCIUM_ION_TRANSPORT
GOBP_NEGATIVE_REGULATION_OF_CALCIUM_MEDIATED_SIGNALING

GOBP_REGULATION_OF_CYTOSOLIC_CALCIUM_ION_CONCENTRATION
GOBP_CYTOSOLIC_CALCIUM_ION_TRANSPORT
GOBP_REGULATION_OF_CALCINEURIN_MEDIATED_SIGNALING
HALLMARK_APICAL_JUNCTION
HALLMARK_APICAL_SURFACE
HALLMARK_APOPTOSIS
HALLMARK_COMPLEMENT
KEGG_BASAL_TRANSCRIPTION_FACTORS
KEGG_GNRH_SIGNALING_PATHWAY
KEGG_PHOSPHATIDYLINOSITOL_SIGNALING_SYSTEM
KEGG_PPAR_SIGNALING_PATHWAY
KEGG_UBIQUITIN_MEDIATED_PROTEOLYSIS

# MSigDB annotations - VASCULAR
GOBP_BLOOD_VESSEL_ENDOTHELIAL_CELL_MIGRATION
GOBP_BLOOD_VESSEL_REMODELING
GOBP_CELLULAR_RESPONSE_TO_VASCULAR_ENDOTHELIAL_GROWTH_FACTOR_STIMULUS
GOBP_ENDOTHELIAL_CELL_DEVELOPMENT
GOBP_ENDOTHELIAL_CELL_MIGRATION
GOBP_ENDOTHELIAL_CELL_PROLIFERATION
GOBP_ENDOTHELIUM_DEVELOPMENT
GOBP_ESTABLISHMENT_OF_ENDOTHELIAL_BARRIER
GOBP_LYMPHANGIOGENESIS
GOBP_LYMPH_VESSEL_DEVELOPMENT
GOBP_MORPHOGENESIS_OF_AN_ENDOTHELIUM
GOBP_NEGATIVE_REGULATION_OF_BLOOD_CIRCULATION
GOBP_NEGATIVE_REGULATION_OF_BLOOD_VESSEL_ENDOTHELIAL_CELL_MIGRATION
GOBP_NEGATIVE_REGULATION_OF_ENDOTHELIAL_CELL_PROLIFERATION
GOBP_NEGATIVE_REGULATION_OF_HEMOPOIESIS
GOBP_NEGATIVE_REGULATION_OF_VASCULAR_ASSOCIATED_SMOOTH_MUSCLE_CELL_PROLIFERATION
GOBP_NEGATIVE_REGULATION_OF_VASCULATURE_DEVELOPMENT
GOBP_POSITIVE_REGULATION_OF_BLOOD_VESSEL_ENDOTHELIAL_CELL_MIGRATION
GOBP_POSITIVE_REGULATION_OF_ENDOTHELIAL_CELL_APOPTOTIC_PROCESS
GOBP_POSITIVE_REGULATION_OF_ENDOTHELIAL_CELL_MIGRATION
GOBP_POSITIVE_REGULATION_OF_ENDOTHELIAL_CELL_PROLIFERATION
GOBP_POSITIVE_REGULATION_OF_HEMOPOIESIS
GOBP_POSITIVE_REGULATION_OF_VASCULAR_ASSOCIATED_SMOOTH_MUSCLE_CELL_PROLIFERATION
GOBP_POSITIVE_REGULATION_OF_VASCULATURE_DEVELOPMENT
GOBP_POSITIVE_REGULATION_OF_VASOCONSTRICTION
GOBP_REGULATION_OF_BLOOD_CIRCULATION
GOBP_REGULATION_OF_ENDOTHELIAL_CELL_MIGRATION
GOBP_REGULATION_OF_HEMOPOIESIS
GOBP_REGULATION_OF_SPROUTING_ANGIOGENESIS
GOBP_REGULATION_OF_VASCULAR_ENDOTHELIAL_GROWTH_FACTOR_RECEPTOR_SIGNALING_PATHWAY
GOBP_REGULATION_OF_VASCULAR_PERMEABILITY
GOBP_REGULATION_OF_VASCULATURE_DEVELOPMENT
GOBP_SPROUTING_ANGIOGENESIS
GOBP_VASCULAR_ASSOCIATED_SMOOTH_MUSCLE_CELL_DIFFERENTIATION
GOBP_VASCULAR_ASSOCIATED_SMOOTH_MUSCLE_CELL_MIGRATION
GOBP_VASCULAR_ASSOCIATED_SMOOTH_MUSCLE_CELL_PROLIFERATION
GOBP_VASCULAR_ENDOTHELIAL_CELL_PROLIFERATION
GOBP_VASCULAR_ENDOTHELIAL_GROWTH_FACTOR_RECEPTOR_SIGNALING_PATHWAY

GOBP_VASCULAR_ENDOTHELIAL_GROWTH_FACTOR_SIGNALING_PATHWAY
GOBP_VASCULOGENESIS
GOBP_CELLULAR_EXTRAVASATION
GOBP_POSITIVE_REGULATION_OF_CELLULAR_EXTRAVASATION
GOBP_REGULATION_OF_CELL_MIGRATION_INVOLVED_IN_SPROUTING_ANGIOGENESIS
HALLMARK_COAGULATION
KEGG_VEGF_SIGNALING_PATHWAY

# MSigDB annotations - WNT PATHWAY
GOBP_CANONICAL_WNT_SIGNALING_PATHWAY
GOBP_NEGATIVE_REGULATION_OF_WNT_SIGNALING_PATHWAY
GOBP_NON_CANONICAL_WNT_SIGNALING_PATHWAY
GOBP_POSITIVE_REGULATION_OF_CANONICAL_WNT_SIGNALING_PATHWAY
GOBP_POSITIVE_REGULATION_OF_WNT_SIGNALING_PATHWAY
GOBP_REGULATION_OF_CANONICAL_WNT_SIGNALING_PATHWAY
GOBP_REGULATION_OF_WNT_SIGNALING_PATHWAY
GOBP_CELL_CELL_SIGNALING_BY_WNT
GOBP_NEGATIVE_REGULATION_OF_CANONICAL_WNT_SIGNALING_PATHWAY
KEGG_WNT_SIGNALING_PATHWAY
