# Supplementary material for: Consensus clustering methodology to improve molecular stratification of non-small cell lung cancer
Source: Sci Rep. 2023 May 12;13:7759. doi: 10.1038/s41598-023-33954-x (PMC10182023; doi:10.1038/s41598-023-33954-x)
Supplement: Supplementary file 2 — Supplementary Information 2. [file 41598_2023_33954_MOESM2_ESM.docx]

**Supplementary Materials**


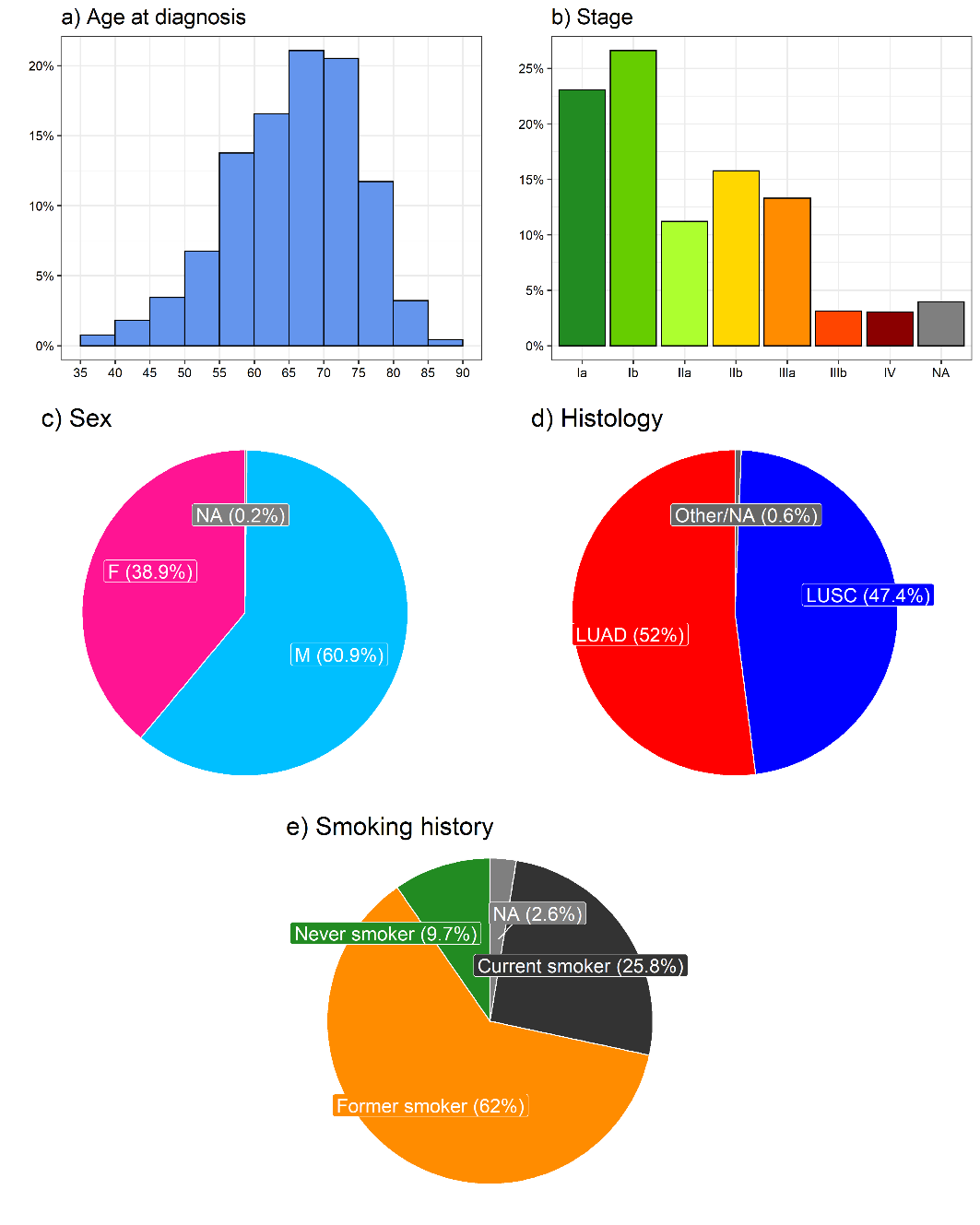


Supplementary Figure 1. Overview of the main clinical variables of the patients enrolled in the clinical study, namely: (a) histogram of age at diagnosis; (b) histogram of clinical staging at diagnosis; (c) gender; (d) histological subtypes; (e) smoking history. Missing values are mostly due to TCGA incomplete clinical data.


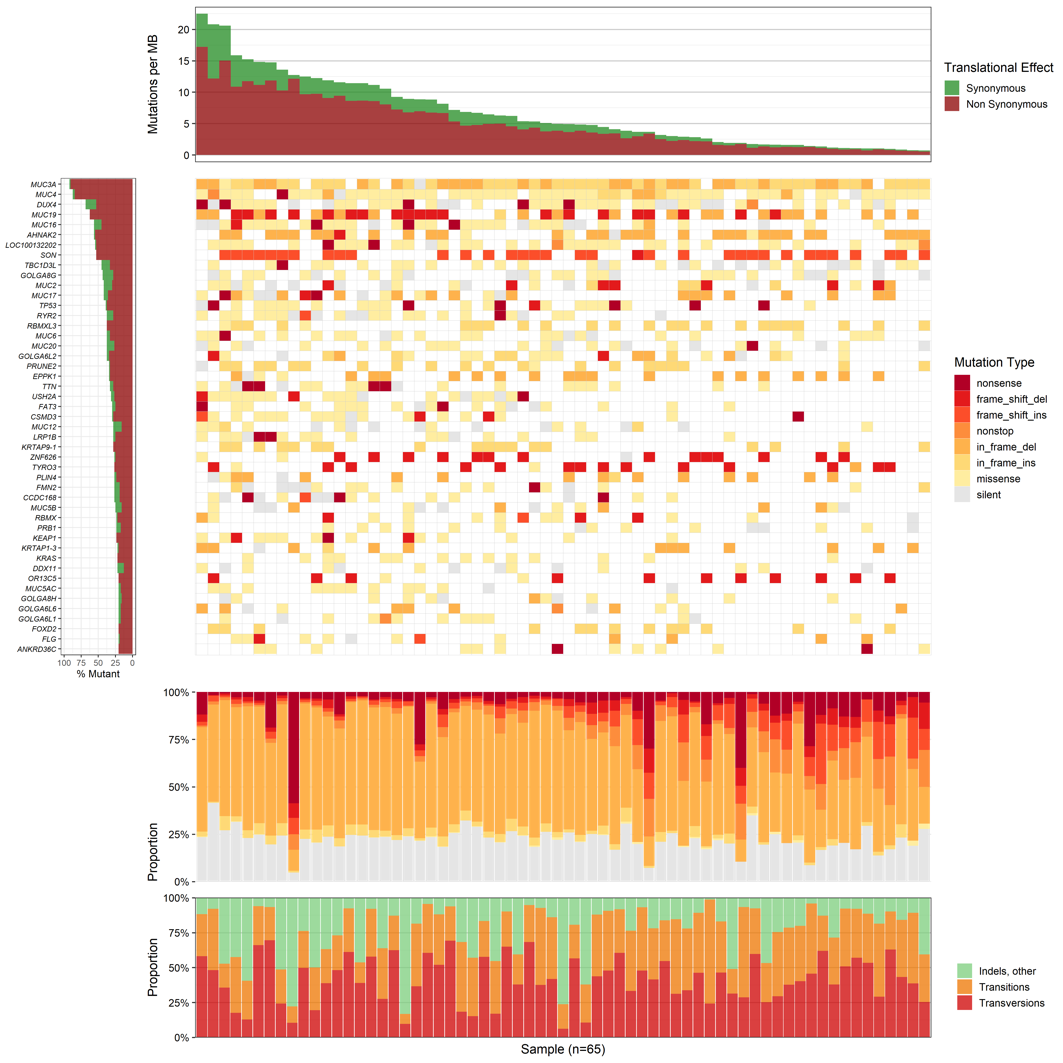


Supplementary Figure 2. Detailed overview of the mutational landscape of patients enrolled in the clinical study. Heatmap: mutation matrix with patients on the columns and top 47 most mutated genes on the rows, colours indicate mutation types prioritised as: nonsense mutations, frameshift deletions, frameshift insertions, nonstop mutations, in-frame deletions, in-frame insertions, missense mutations and silent mutations; the proportion of each mutation type is reported in the panel below the matrix. Patients and genes are ordered by decreasing mutational burden and decreasing percentage of mutated samples respectively represented on top and left panels, colours refer to transcriptional effect. Bottom panel: proportion of indels and SNV transitions/transversions per each patient.


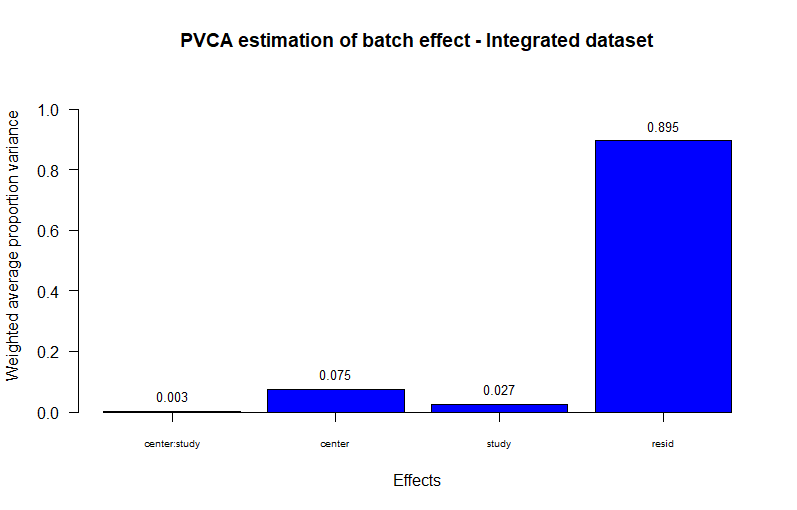


Supplementary Figure 3. Variance explained by each possible source of batch-effect quantified via PVCA. “Study” refers to DEFLeCT/TCGA samples, which explains 2.7% of the total variance; “center” refers to the hospital were each patient has been enrolled, which explains 7.5% of the total variance.

Supplementary Table 1. Percentage of each histological subtype (LUAD/LUSC) assigned to each cluster as a function of the final number of cluster (k) considered. Last rows indicate the values of Silhouette scores. Best configurations are highlighted in bold.

|  | k=3 | **k=4** | k=5 | k=6 |
| --- | --- | --- | --- | --- |
| % LUAD A | 5.7 | **93.1** | 87.9 | 9.4 |
| % LUAD B | 78.9 | **86.4** | 90.6 | 56.1 |
| % LUAD C | 92.8 | 8.1 | 3.3 | 93.7 |
| % LUAD D | - | 1.4 | 1.8 | 3.1 |
| % LUAD E | - | - | 27.5 | 89.6 |
| % LUAD F | - | - | - | 0.0 |
| % LUSC A | 94.3 | 6.9 | 12.1 | 90.6 |
| % LUSC B | 21.1 | 13.6 | 9.4 | 43.9 |
| % LUSC C | 7.2 | **91.9** | 96.7 | 6.3 |
| % LUSC D | - | **98.6** | 98.2 | 96.9 |
| % LUSC E | - | - | 72.5 | 10.4 |
| % LUSC F | - | - | - | 100.0 |
| Silhouette Index | -0.03 | **-0.02** | -0.04 | -0.06 |


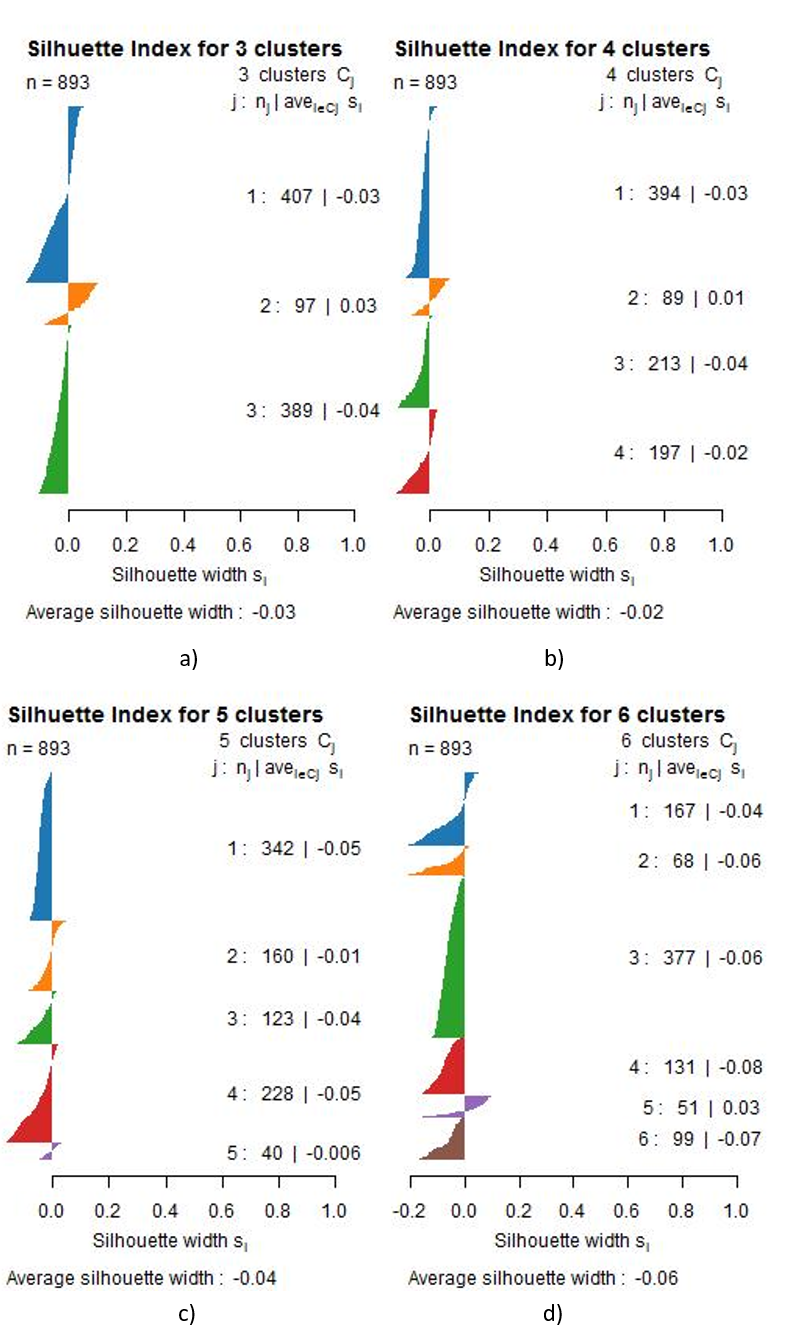


Supplementary Figure 4. Summary of Silhouette index analysis for the 4 final number of clusters considered (panels). Bars indicate Silhouette value for each individual sample, colours refer to clusters. Average Silhouette width for each configuration is reported on the bottom.


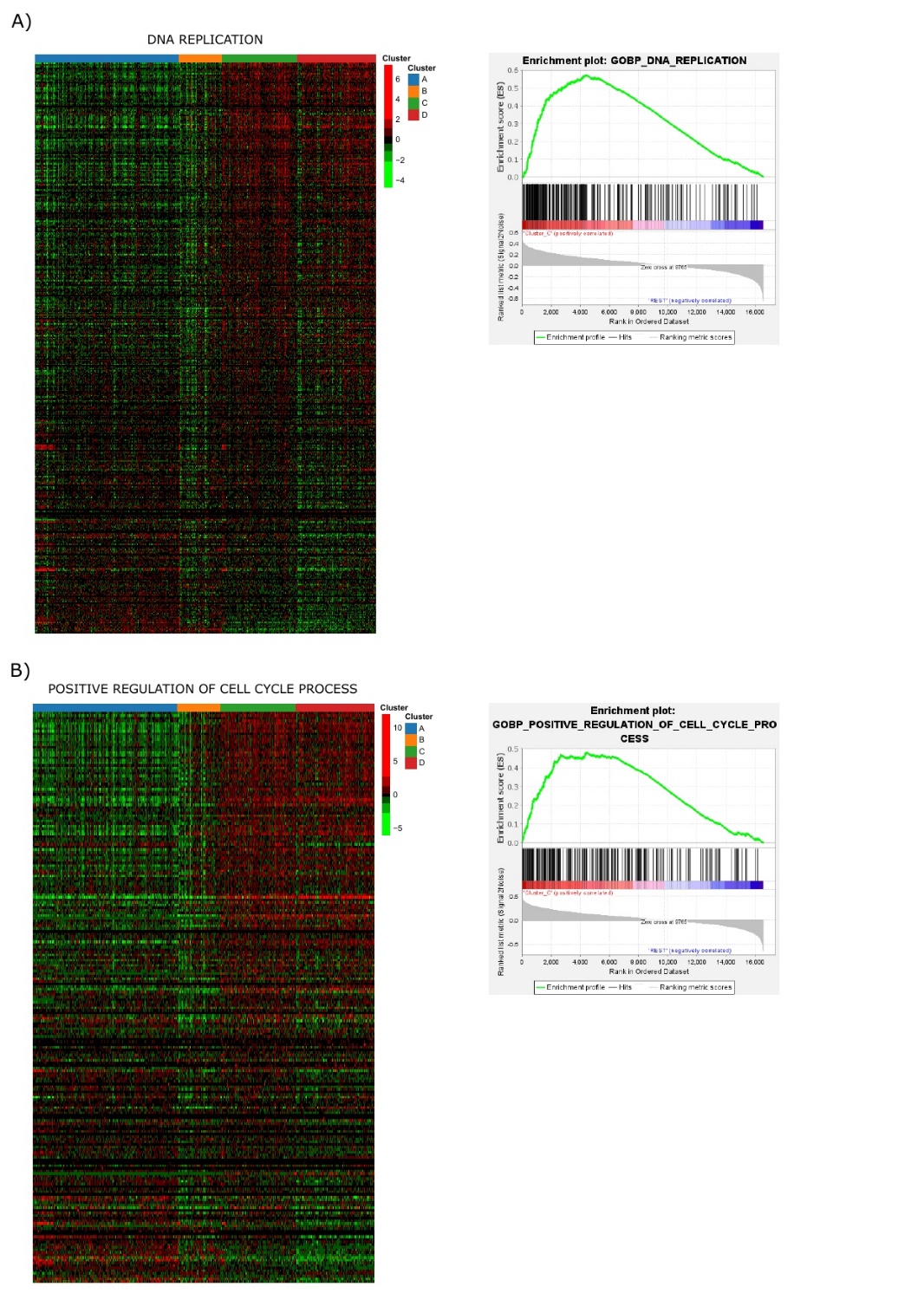


Supplementary Figure 5. Representative gene sets from the MSigDB found enriched in cluster C (versus the other clusters) with FDR q-value<0.1. Each panel reports the GSEA enrichment plot (right) and the corresponding heatmap of the genes belonging to the selected gene set (left), columns of the heatmap are grouped by cluster. Panels refer to: A) DNA repair (NES=1.9, gene set size: 438, core enrichment size: 218), B) Positive regulation of cell cycle process (NES=1.83, gene set size: 217, core enrichment size: 106).


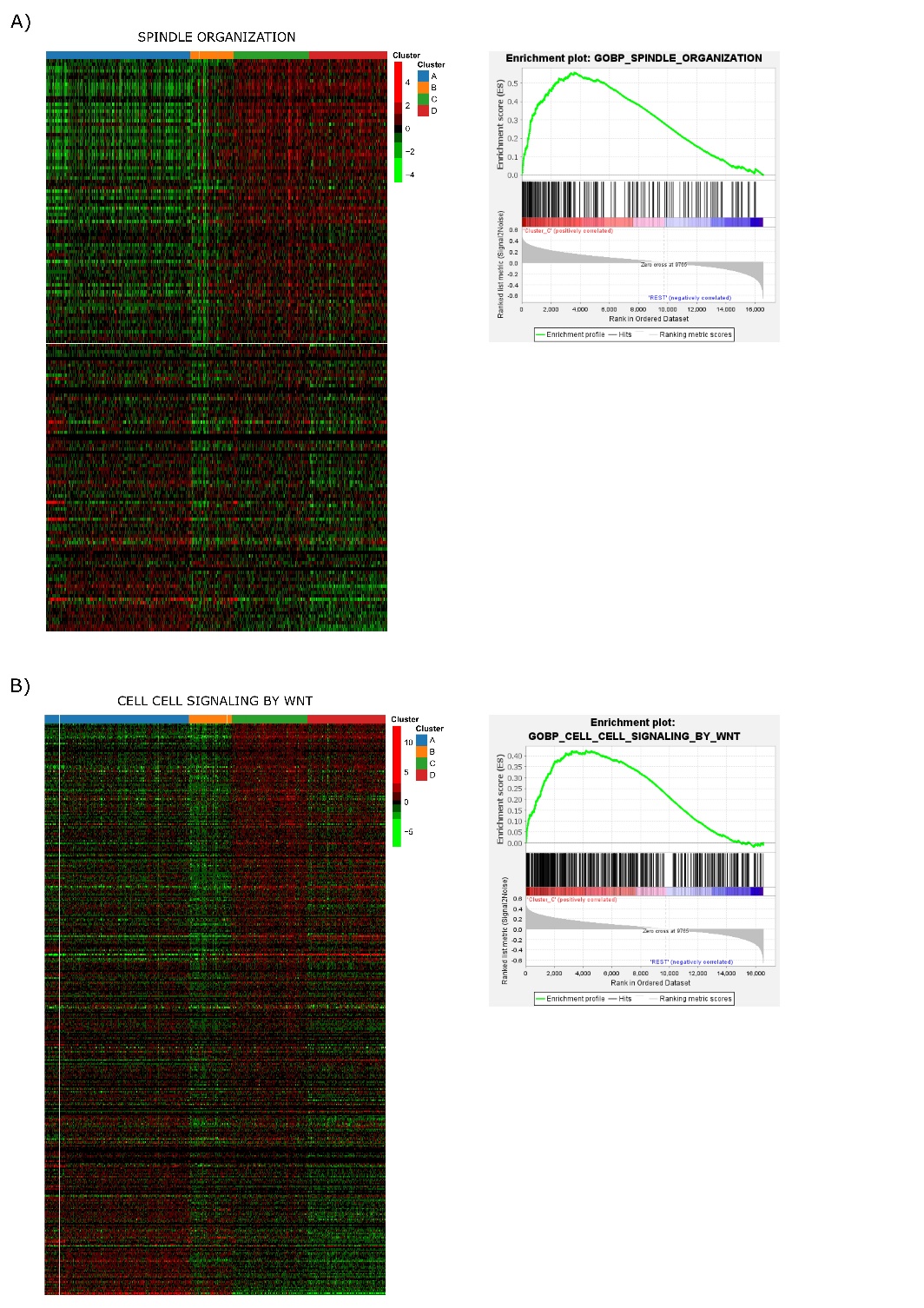


Supplementary Figure 6. Representative gene sets from the MSigDB found enriched in cluster C (versus the other clusters) with FDR q-value<0.1. Each panel reports the GSEA enrichment plot (right) and the corresponding heatmap of the genes belonging to the selected gene set (left), columns of the heatmap are grouped by cluster. Panels refer to: A) Spindle organisation (NES=1.95, gene set size: 171, core enrichment size: 89), B) Cell cell signaling by WNT (NES=1.84, gene set size: 412, core enrichment size: 177).


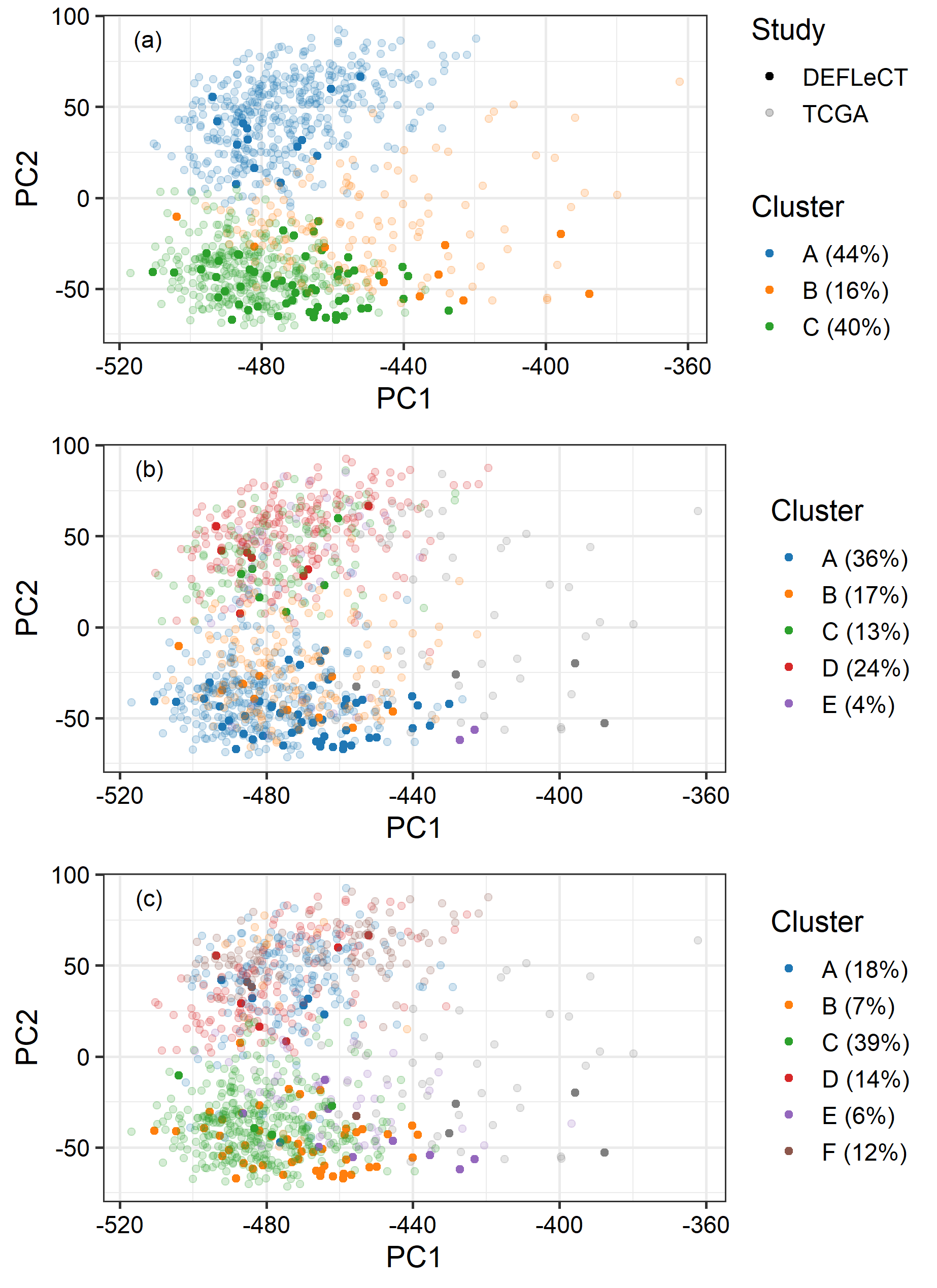


Supplementary Figure 7. First two principal components of the gene expression profiles of TCGA (transparent) and DEFLeCT (opaque) samples obtained via PCA of the log2-scaled TPM. Colours indicate the molecular subgroups identified via consensus clustering; relative abundance of each subgroup is reported in the legend. Panels (a), (b) and (c) refers to the final number of clusters considered (3, 5 and 6 respectively).


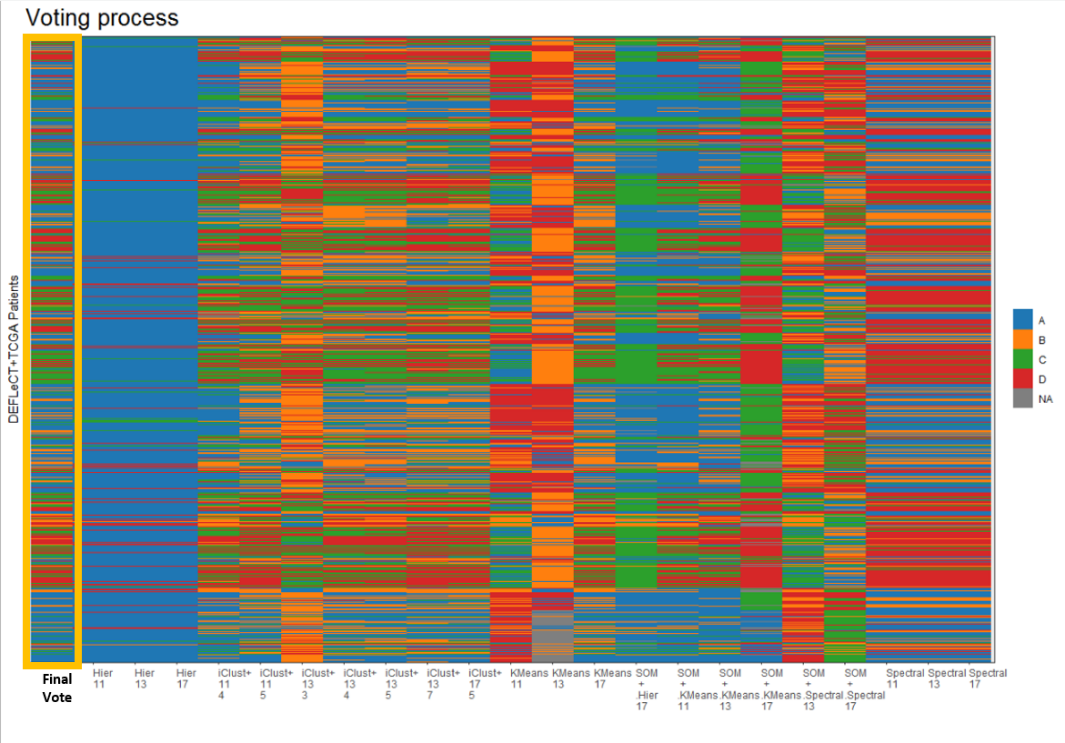


Supplementary Figure 8. Visual representation of consensus clustering (majority voting algorithm). The heatmap represent the vote of each independent learner (columns) for each sample of the dataset (rows). Final labels are represented and highlighted in the first column. Colours of the heatmap refer to cluster labels.

Supplementary Table 2. Results of $\chi^{2}$ test assessing whether the main clinical variables are overall significatively differently distributed among identified subgroups

| Variable | $\chi^{2}$ | p-value |
| --- | --- | --- |
| Histological subtype | 699.8 | <0.001*** |
| Gender | 75.2 | <0.001*** |
| Age (2 classes) | 17.5 | 0.0016** |
| Smoking history | 42.4 | <0.001*** |
| Stage (4 classes) | 66.3 | <0.001*** |
